# Evaluating public interest in herpes zoster in Germany by leveraging the internet: a retrospective search data analysis

**DOI:** 10.1186/s12889-023-16463-4

**Published:** 2023-08-15

**Authors:** Alphina Kain, Linda Tizek, Hannah Wecker, Fabian Wallnöfer, Tilo Biedermann, Alexander Zink

**Affiliations:** 1 School of Medicine, Department of Dermatology and Allergy, Technical University of Munich, Biedersteiner Str. 29, 80802 Munich, Germany; 2 Division of Dermatology and Venereology, Department of Medicine Solna, Karolinska Institutet, Stockholm, Sweden

**Keywords:** Herpes zoster, Postherpetic neuralgia, Search engine, Internet, Infodemiology

## Abstract

**Background:**

Herpes zoster (HZ) and its complication postherpetic neuralgia (PHN), whose incidence are both expected to increase with an ageing population, have demonstrated high costs on healthcare systems and burden on individual quality of life. Previous studies have shown the possibility of assessing public interest in a disease and factors that influence search behaviour using internet search data. The aim of this study was to analyze internet search data for HZ in Germany to evaluate public interest in the disease and relevant influential temporal and geographic factors that modify search behavior.

**Methods:**

Google Ads Keyword Planner was used to generate a list of HZ-related keywords including their search volume for Germany as a whole and its sixteen federal states from October 2016 to September 2020. All keywords were qualitatively categorized, and changes over time and correlations with population density, physician density, and vaccination rates were assessed using Welch’s ANOVA, Bonferroni correction for post-hoc analyses, and Pearson’s correlation.

**Results:**

A total of 1,651 relevant keywords with a search volume of 20,816,210 searches were identified. Overall, national search volume increased each year of the study period with a peak in August 2020. More than half of the total search volume related to general queries (55.1%). The highest average monthly search volumes were observed in the states of Hamburg, Saarland, and Bremen. Average monthly search volume showed strong positive correlations with population density (*r* = .512, *p* = .043) and a strong negative correlation with the number of inhabitants per working physician (*r* = -.689, *p* = .003).

**Conclusions:**

The study demonstrated that evaluating internet search data is a viable method for assessing public interest in HZ, thereby identifying areas of unmet need to support targeted public health campaigns.

**Supplementary Information:**

The online version contains supplementary material available at 10.1186/s12889-023-16463-4.

## Background

Herpes zoster (HZ), an infectious disease caused by a reactivation of the varicella zoster virus (VZV) in sensory ganglia [1]. The disease typically occurs after a period of dormancy following primary infection with the virus, and it is characterized by a painful and blistering dermatomal rash [1]. The most frequently affected dermatomes in descending order are thoracic, cranial, cervical, and lumbosacral dermatomes [2]. More commonly known as shingles, it has an overall lifetime risk of 20 to 30%, increasing to 50% in individuals aged 85 [3]. Particularly individuals aged 65 and older are more likely to develop HZ, which is believed to be caused by an age-related decline in T-cell immune response [4]. Other causes for immunodeficiency, like disease (e.g., lymphoma) and immunosuppressive drugs, also increase the risk for HZ [5]. Additionally, some studies have identified the role of increased ambient temperature and ultraviolet (UV) radiation in raising the HZ incidence [6, 7].

Therapy includes a combination of antiviral medication, pain management, and treatment of possible complications, like ocular damage in zoster ophthalmicus [5]. Other potential complications of the disease include motor paralysis, meningoencephalitis, and most commonly, postherpetic neuralgia (PHN) [8]. The risk for complications increases with age, with up to 70% of individuals 60 years and older experiencing PHN after HZ compared to only 34% of the general population [9]. HZ treatment should also be administered within the first 72 hours after the initial rash outbreak to minimize complications, although delays in accessing medical care because of appointment waiting times or atypical disease presentation may increase the risk of complications [10]. Particularly insurance status and regional differences in access to medical care influence appointment waiting times in Germany [11]. The loss of quality-adjusted life years (QALY), a measure of disease burden, is reported to be between 3,065 and 24,094 QALY for HZ in Germany alone each year [12]. HZ symptoms and its complications have demonstrated substantial influence on individual quality of life [13]. In Germany, patients with HZ and PHN as well as their relatives have reported significant physical and psychological impairment because of these diseases [14]. The burden of HZ is expected to increase with population ageing becoming a growing global phenomenon. However, two vaccines are currently available for HZ, with the newer recombinant vaccine being made a recommended vaccine for people 60 years and older by the Standing Committee on Vaccination (STIKO) of the Robert Koch Institute in Germany [15]. The older live attenuated vaccine was approved earlier in Germany in 2013, but it was not made a recommended vaccine because of its reduced efficacy with increasing age and increasing time since vaccination [16]. As of 2022, HZ vaccination rates for eligible publicly insured persons are 11.5% for one dose and 7.7% for both recommended doses in Germany [17], with large parts of the potentially affected population remaining vulnerable to HZ and its complications.

In Germany, where the Google search engine has a 95% market share and 9 out 10 of people use the internet, 57% of people use the internet for health-related information at least once in 12 months [18–20]. Data like internet search volume have already been leveraged to analyze interest, identify areas of unmet medical need, and seasonal variability for several dermatological conditions [21–23]. Since the advent of the COVID-19 pandemic, there has also been a rise in the number of patients missing medical appointments [24]. As Wang et al. describe, patients with HZ who miss medical appointments are likely to have a higher risk of developing complications [24]. Reduced access to clinical settings, both voluntarily and involuntarily, reflects the growing relevance of alternate sources like the internet for health information. Additionally, regional differences in healthcare access as well as demographic differences between rural and urban areas in Germany have influenced utilization of medical services [25]. Rural regions in Germany are more likely to face physician shortages [26] and regions with higher physician densities see a higher use of outpatient services [27]. The aim of this study was to therefore analyze internet search data for HZ in Germany and its sixteen federal states to evaluate public interest in HZ and relevant influential temporal and geographic factors to identify areas of unmet need.

## Methods

### Study design

In this retrospective study, Google Ads Keywords planner was used to determine the web search volume for keywords related to HZ from October 2016 to September 2020. While originally developed for marketing purposes, the software has also seen applications in scientific investigations [28]. Google Ads Keywords Planner provides a list of relevant search terms and their search volume for the last 48 months after entering a particular word or phrase, which in this study were “herpes zoster” and the German layman’s term for shingles (“Gürtelrose”). Data were generated by the program for Germany as a whole and its 16 federal states. Only data for searches made in Germany and with German set as the preferred language were considered. As data for the study used publicly available search terms, institutional review board approval was not required, and informed consent was not applicable.

### Classification and statistics

All identified keywords were qualitatively assessed and classified as either relevant or irrelevant keywords. Search terms that did not mention HZ or were inapplicable were excluded from further analyses. Search terms that did not mention HZ, were inapplicable because they, for example, related to only animals, or were repetitions of the same keywords were excluded from further analyses. The remaining relevant search terms were classified into 11 categories: (1) *general* (e.g., “shingles”), (2) *localization* (e.g., “shingles face”), (3) *symptoms and severity* (e.g., “shingles symptoms”), (4) *contagiousness* (e.g., “is shingles contagious”), (5) *therapy* (e.g., “shingles treatment”), (6) *causes* (“chickenpox shingles”), (7) *patient characteristics* (e.g., “shingles children”), (8) *complications* (e.g., “postherpetic neuralgia”), (9) *information* (e.g., “shingles wikipedia”), (10) *vaccine* (e.g., “vaccination shingles”), and (11) *other diseases* (e.g., “shingles hiv”). The eleven categories were continuously formed from the data. In this process, search terms were analyzed one at a time and appropriate categories were formed. If a new search term did not meet the inclusion criteria for an existing category, a new category was formed. If there were only few search terms for certain categories, categories were combined or assigned to the general category, where applicable. Subcategories for categories were formed for further insight into search behavior. However, as keywords that matched several criteria were assigned to multiple categories and subcategories, cumulative percentages may have exceeded 100%.

To identify seasonal differences in search behavior, the average monthly search volume for spring (March to May), summer (June to August), autumn (September to November), and winter (December to February) in Germany was calculated. Data for the year 2016 were excluded when analyzing search volume over time due to the low number of observations. To assess differences in search volume between the German federal states, the search volume was calculated per 100,000 inhabitants for all federal states and Germany as a whole [29].

After the assumptions of ANOVA were not met, Welch’s ANOVA was conducted to evaluate changes over time and regional differences. The assumptions were checked with Levene’s test for variance homogeneity and Q-Q Plots for normal distribution. In addition, independence was assumed. The Bonferroni correction was used for post-hoc analyses of seasonal and geographic differences. To evaluate the role of regional differences on search interest, Pearson’s correlation coefficient was used to analyze the relationship between search volume and vaccination rates, population density, and the number of inhabitants per working physician for each federal state [17, 29, 30]. *P*-values < .05 were considered statistically significant. For all statistical analysis, the open-source software JASP version 0.16.1 was used (University of Amsterdam, Netherlands) [31].

## Results

For Germany, 1,694 keywords related to HZ were identified, of which 43 were considered irrelevant and were excluded from analyses. The remaining 1,651 keywords had a total search volume of 20,816,210 searches, translating to 25,033 searches per 100,000 inhabitants. The keyword with the highest search volume was the German layman’s term for shingles (“Gürtelrose”) followed by “shingles contagious” (“Gürtelrose ansteckend”) and “shingles symptoms” (“Gürtelrose symptome”).

### Search volume over time

**Fig. 1:**
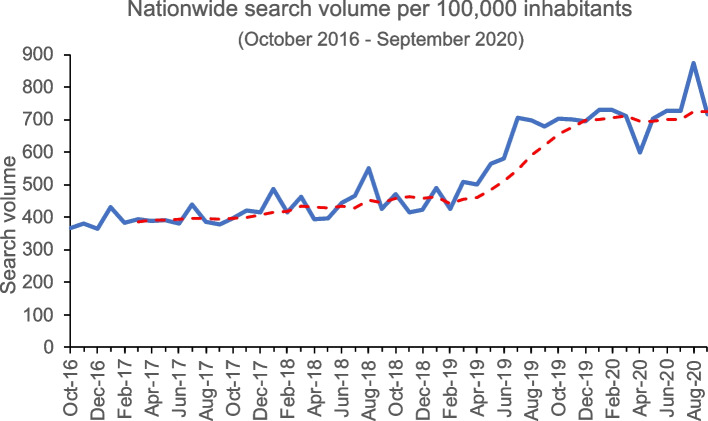
Nationwide search volume for shingles-related Google searches in Germany from October 2016 to September 2020. The moving average trendline is represented by the dashed red line

The average search volume per month per 100,000 inhabitants increased during the study period (Fig. 1). Differences between the years were found (*p* < .001). In 2017, an average of 400.7 (± SD 20.7) searches per month per 100,000 inhabitants were conducted. This increased to 445.8 (± SD 44.7, *p* = .0644) searches per month in 2018, to 604.0 (± SD 103.9, *p* < .001) in 2019, and to 724.0 (± SD 69.6, *p* < .001) in 2020. The average search volume in 2020 was higher than in 2019 (*p* = .001). The lowest search volume was observed for December 2016 and the highest for August 2020 (Fig. 1). ,Differences in search volume by season were observed, but these were shown to not be significant (spring: 500.9 ± SD 119.9; summer: 581.2 ± SD 161.7; autumn: 504.7 ± SD 147.0; winter: 499.3 ± SD 137.1; *p*=.53).

### Categories

The category with the highest overall search volume was the category *general*, with 11,475,560 (55.1%) searches, followed by the categories *localization* and *symptoms and severity*, with 3,567,790 (17.1%) and 2,130,210 searches (10.2%), respectively (Table 1). Searches related to *other diseases* and a shingles *vaccine* had the lowest search volumes, with 0.4% and 0.8% of the total search volume, respectively.

**Table 1:** Keyword categories, their search volume, and their percentage* of the total search volume for the Google search volume of HZ-related keywords in Germany

**Category**	**Number of keywords**	**Search volume**	**Percentage of total search volume**^a^
General	132	11,476,110	55.1%
Localization	558	3,567,790	17.1%
Symptoms and severity	334	2,130,210	10.2%
Contagiousness	136	1,837,870	8.8%
Therapy	198	855,570	4.1%
Causes	128	518,330	2.5%
Patient characteristics	137	510,470	2.5%
Complications	139	465,090	2.2%
Information	122	252,340	1.2%
Vaccine	19	163,740	0.8%
Other diseases	66	76,630	0.4%

^a^As keywords were assigned to multiple categories if they met several criteria, the sum of keywords for each category and the sum of the search volume for each category exceed the total number of keywords (*n*=1,651) and overall search volume (*n*=20,816,210), respectively. The percentages of the total search volume were calculated by dividing the search volume per category by the total search volume. The total percentage therefore exceed 100%.

**Fig. 2:**
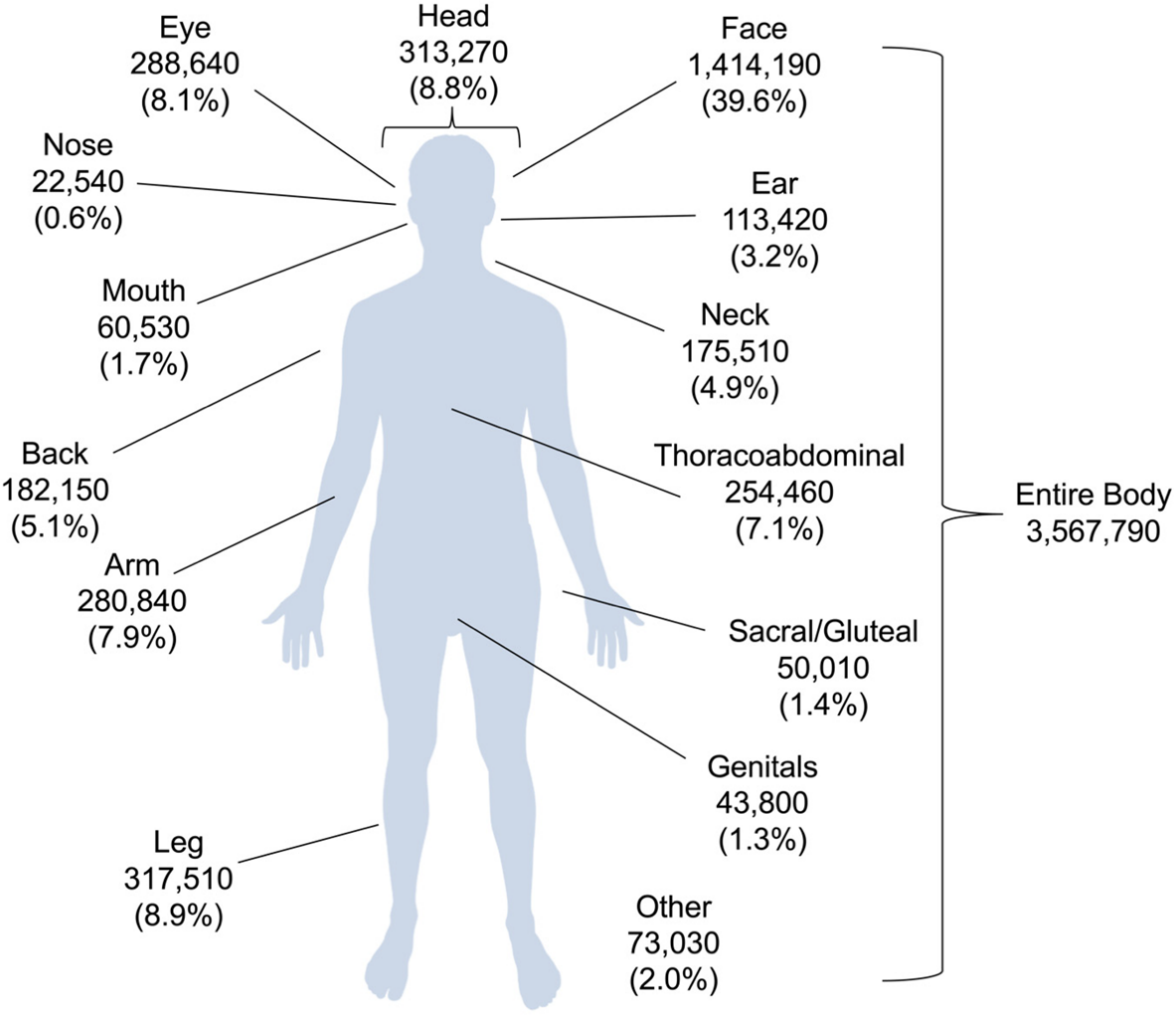
Search volume of shingles-related web searches in Germany from October 2016 to September 2020 grouped according to body localizations

In the *localization* category, most searches related to the face (39.6%), followed by the legs (8.9%) and head in general (8.8%) (Fig. 2). In the category *symptoms and severity*, 41.2% of searches were general queries, with searches about pain (17.1%) and skin changes (13.2%) being the two most searched for specific symptoms (Table 2). After general searches, searches for alternative therapy (20.4%) had the highest search volume in the category *therapy*. In the *causes* category, nearly half of searches related to chickenpox or varicella (46.2%) followed by general queries (38.2%). Regarding patient characteristics, most searches were about children (52.9%) and pregnant individuals (34.2%). Searches about PHN constituted the majority of searches in the *complications* category (59.8%).

**Table 2:** Google search volume for select categories and their five largest subcategories with respective percentages for HZ-related keywords in Germany

**Category**	**Search volume**	**Top 5 subcategories (by search volume)**^a^				
Localization	3,567,790	Face *n*=1,414,190; 39.6%	Leg *n*=317,510; 8.9%	Head *n*=313,270; 8.8%	Eye *n*=288,640; 8.1%	Arm=280,840; 7.9%
Symptoms and severity	2,130,210	General *n*=877,130; 41.2%	Pain *n*=364,110; 17.1%	Skin/Blisters *n*=280,150; 13.2%	Absence *n*=238,870, 11.2%	Other *n*=41,500; 1.9%
Therapy	855,570	General *n*=560,120; 65.5%	Alternative *n*=174,750; 20.4%	Medication *n*=68,580; 8.0%	No Therapy *n*=17,880; 2.1%	Physician *n*=17,550; 2.1%
Causes	518,330	Chickenpox/Pathogen *n*=239,670; 46.2%	General *n*=185,450; 35.8%	Stress *n*=33,310; 6.4%	Mental/Spiritual *n*=30,930; 6.0%	Other *n*=14,730; 2.8%
Patient characteristics	510,470	Children *n*=269,830; 52.9%	Pregnancy *n*=174,550; 34.2%	Adults *n*=26,120; 5.1%	Elderly *n*=23,760; 4.7%	Young people *n*=9,580; 1.9%
Complications	465,090	Postherpetic neuralgia *n*=278,030; 59.8%	General *n*=100,610; 21.6%	Death *n*=49,530; 10.6%	Other *n*=25,640; 5.5%	Scarring *n*=11,280; 2.4%

^a^As keywords were assigned to multiple subcategories if they met several criteria, the sum of keywords and the sum of the search volume for each subcategory may exceed the total number of keywords and search volume for each category, respectively. Percentages for the subcategories were calculated by dividing the search volume per subcategory by the category search volume. The total percentages therefore may exceed 100%.

### Comparison between the 16 German federal states

**Fig. 3:**
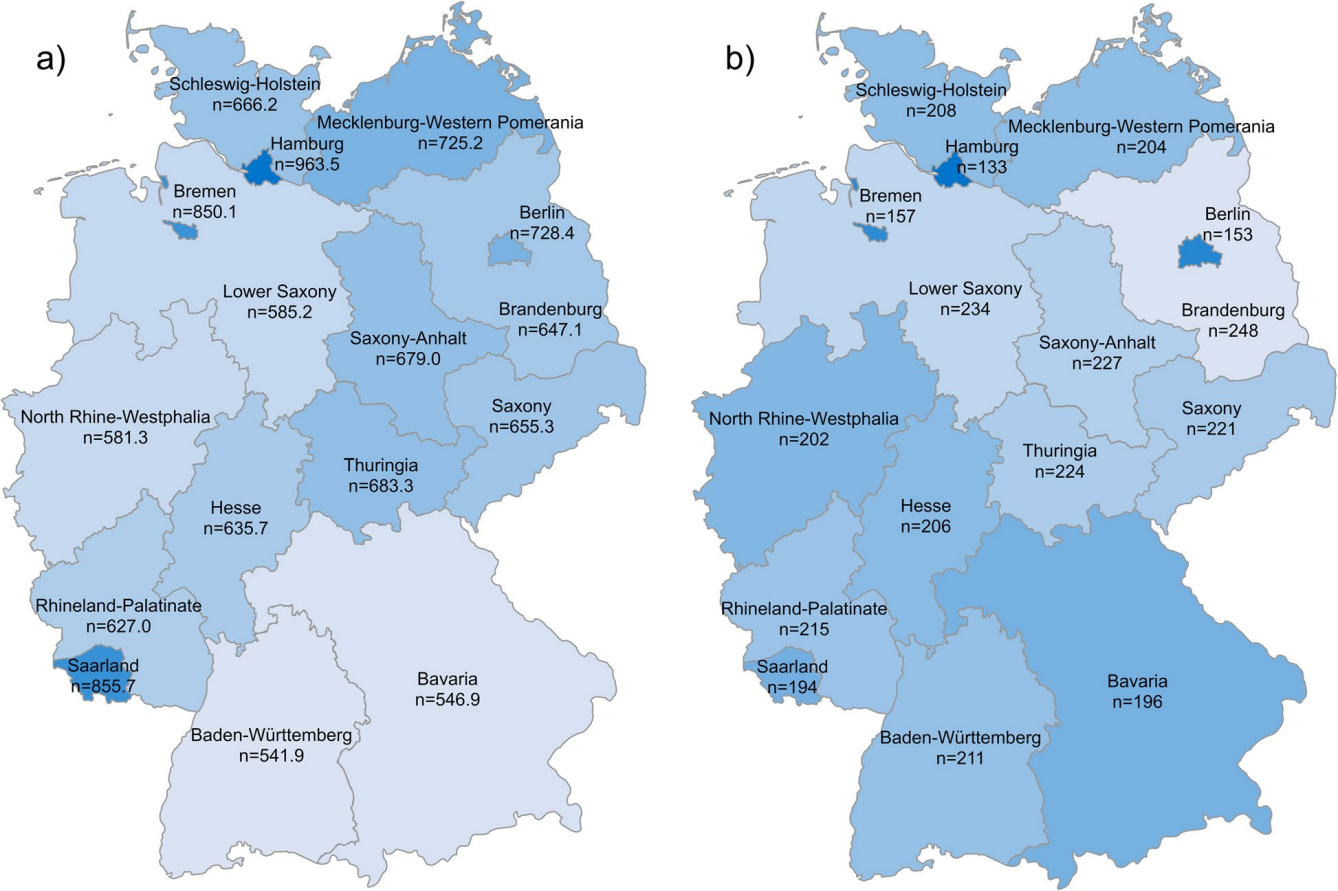
Colour-coded maps of the sixteen federal states of Germany showing a) the average monthly search volume per 100,000 inhabitants for shingles-related keywords and b) the physician density in each federal state expressed as the number of residents per each working physician. A darker blue colour indicates a higher search volume per capita as well as a higher physician density

For Germany, the average search volume per month was 521.5 (± SD 141.2) searches per 100,000 inhabitants (Supplementary Table 1). Among the federal states, the highest monthly search volume per 100,000 inhabitants was observed for Hamburg (963.5 ± SD 211.7) followed by Saarland (855.7 ± SD 188.7) and Bremen (850.1 ± SD 147.0) (Fig. 3). The search volume for these three states was significantly higher than the observed lowest monthly search volume per 100,000 inhabitants in Baden-Württemberg (541.9 ± SD 138.3) followed by that in Bavaria (546.9 ± SD 144.1) and North Rhine-Westphalia (581.2 ± SD 152.8).

A high positive correlation was demonstrated between the average monthly search volume per 100,000 inhabitants and population density (*r* = .512, *p* = .043). Inversely, there was a strong negative correlation between the average monthly search volume and the number of inhabitants per working physician in each federal state (*r* = -.689, *p* = .003) (Fig. 3). Vaccination rates for both one or two doses of the approved HZ vaccine correlated negatively with average monthly search volume, but this correlation did not reach significance (one dose: *r* = -.141, *p* = .603: two doses: *r* = -.171, *p* = .526).

## Discussion

The aim of this study was to analyze the web search volume for shingles in Germany as a whole and its sixteen federal states, focusing on seasonal and geographic factors that modulate search behavior. Overall, the results demonstrated an increasing interest in HZ in Germany during the 48-month study period, with a total of 20.8 million searches conducted. Comparable studies in Germany reported 8.8 million searches for atopic dermatitis, 11.4 million searches for scabies, 13.7 million searches for pruritus, and 19.8 million searches for skin cancer [21–23, 28]. This high search volume for HZ, despite the fact that the disease primarily affects older individuals and that internet use is more common in younger people, underlines the relevance of the internet as a source of health information for shingles [32].

The outreach of public health campaigns and public response to health policies may be monitored using online search data. The first substantial increase in search volume, observed during the second half of 2018, occurred after the authorization of the recombinant HZ vaccine by the EMA in March 2018 [33]. Similarly, the peak in search volume in May 2019 was preceded by a sharp increase in March 2019, when German public health insurance coverage was expanded to include the costs of this vaccine [34]. The need for increased awareness about the availability of a shingles vaccine is underlined by the low search volume for vaccines compared to the overall search volume. With only 19 keywords, vaccine-related search volume comprised less than 1% of the total search volume. Future studies should further evaluate the relationship between vaccination rates and internet search volume, particularly as vaccinations have become a major health topic of online discourse in the last years.

Several studies have demonstrated changes in patient behavior in accessing medical care and health information since the COVID-19 pandemic. Waiting times during the pandemic decreased, which researchers attributed to an increased reluctance to visit medical practices out of fear of COVID-19 infection [11]. Guzman and Barbieri reported on decreased web search volumes for several dermatological conditions and cosmetic procedures after lockdown announcements in 2020, with only data for general dermatological conditions reaching pre-pandemic levels during the social distancing period [35]. The search volume for HZ, however, showed a significant increase from 2019 to 2020, which appears to be part of overall trend of increasing search volume during the study period. Furthermore, studies have reported on an increased risk for developing HZ after a COVID-19 infection, which may be reflected in the increase in search volume from 2019 to 2020 [36]. Despite the seemingly opposing behaviors in online search interest, our findings may be in line with those of Guzman and Barbieri, who argued that the return to baseline for the search volume of general dermatological conditions may reflect the growing relevance of the internet for health information during a time where access to in-person medical care is limited [35].

There was no seasonality observed for HZ web search volume, although seasonality for HZ incidence has been reported in one study from Japan [37]. Other studies have identified the role of UV radiation and higher ambient temperature as risk factors for HZ, but the influence of these risk factors on web search data was not demonstrated in this study [6, 7]. As these studies used medical records data, web search data may not be an accurate proxy for disease incidence when analyzing the relationship between HZ and weather.

After general keywords, most searches related to HZ localizations. The face, head in general, and eyes were among the five most searched for body localizations. In contrast, more than half of HZ patients have symptoms in thoracic dermatomes, with trigeminal involvement in less than 20% of cases, followed by cervical, lumbar, and sacral involvement in decreasing order [2]. The greater disease burden for HZ with cranial involvement as well as higher disease visibility because of facial involvement may, however, explain the increased search volume for these predilection sites. Patients with trigeminal involvement had a higher risk of HZ complications, with zoster ophthalmicus increasing the risk 7-fold when compared to the risk associated with thoracic involvement [38]. PHN, which was the most searched for complication in our study, is the most frequently occurring complication, followed by ocular complications and facial palsies [39]. Similar research on psoriasis web search data has demonstrated that harder-to-treat areas were searched more frequently than typical predilection sites, which may be a pattern also observed for HZ-related search behavior [40].

While HZ can occur in children and young adults, it most commonly affects the elderly, with patients 60 years and older comprising half of all cases [41]. In contrast, nearly 90% of web searches about *patient characteristics* related to children or pregnancy. This suggests a lack of readily available information on typically less-affected demographics like young adults and children, particularly when considering the contagious nature of VZV [42]. Queries like “shingles contagious baby” are therefore relevant for both parents and grandparents who may be concerned about possibly infecting at-risk individuals with VZV.

After general therapy inquiries, most searches regarding therapies were for alternative treatment options. As people often require a prescription for most medication to treat HZ, particularly those individuals with persistent pain, searches for alternative therapies may reflect a lack of access to medical care [4]. Searches for alternative therapy may also indicate dissatisfaction with available treatments. In a French study, 15% of patients with acute HZ and 18% of patients with PHN used complementary medicine to manage their symptoms [43]. Future studies should investigate the reasons for interest in alternative medicine to treat HZ, particularly to identify possible barriers to medical care and dissatisfaction with treatment options.

The study demonstrated that regional differences influenced web search behavior, with a high positive correlation observed between population density and average monthly search volume. First, these differences in search volume may the result of regional differences in internet access. In 2018, only approximately 50% of residents in rural Germany had access to broadband internet with 50 Mbps compared with 93.5% in urban areas [44]. Individuals living in areas with adequate internet access may be more likely to use the internet for health information. These differences in search volume may be attributed to demographic differences, with research demonstrating that residents of rural areas in Germany are on average older than those of urban areas and that they use the internet less frequently [45]. However, the accessibility of medical care may also influence search behavior, as suggested by the correlation between search volume and physician density. Rural regions in Germany are more likely to face physician shortages [26]. Although appointment waiting times during the COVID-19 pandemic decreased, patients in regions with an oversupply of physicians had shorter waiting times for medical appointments [11]. Considering that studies have often reported no correlation between HZ incidence and urban/rural settings, the lack of search interest in less densely populated regions may indicate the need for targeted awareness campaigns for higher-risk populations in rural areas [46]. No notable relationship was observed between vaccination rates and search volume, which may be explained by the low prevalence of vaccine-related search volume in the dataset as well as low vaccination rates because of supply-chain issues [17].

There are a few study limitations. First, Google does not provide demographic information about its search engine users with Google Ads Keywords Planner. Definitive conclusions cannot be drawn about the evaluated population. Specific demographic subgroups therefore cannot be studied using this method. The possible underrepresentation of elderly populations may be seen as a limitation of the study. However, the percentage of internet users among the elderly is continuously increasing so that this limitation may soon be negligible [32]. Limiting the preferred language to German may have also excluded data from resident populations that do not speak German. Web search data may also be a poor proxy for disease incidence, as data from individuals who are not patients are also included in the study (e.g family, friends, or researchers), although this presents a strength of the study by providing insight into the interests of people indirectly affected by the disease. Furthermore, the monthly search volumes are only estimates provided by the software’s algorithm, with additional outside verification of the actual search volume not being possible. The observed increasing search volume for HZ keywords during the study period may also only represent the increase in online search queries overall [47], although further data from Google are needed to verify this. The software also restricted data generation to data from the prior 48 months, which may have limited comparisons in search volume between the observed years, as not all years were equally represented. Despite 95% of the German population using Google as its preferred search engine, the possibility of a selection bias remains [19]. Google’s auto-fill function, whereby more frequently searched for keywords are suggested as users type, may be a source of bias in search behavior.

## Conclusions

Evaluating internet search volume has shown to be a promising and viable method for assessing public interest in shingles. The study results demonstrated that HZ-related internet search volume has continuously increased during the study period and that regional factors like population and physician density influence search behavior. Of particularly interest was HZ with trigeminal involvement, possibly mirroring the increased burden of disease for these localizations. The overall increasing search volume underlines the relevance of the internet for health information when access to medical care may be limited, as demonstrated by changes in health seeking behavior during the COVID-19 pandemic as well as regional differences in healthcare utilization. Particularly as the disease incidence and share of older internet users are expected to grow, web search data can provide further insight into HZ to support targeted public health awareness programs in areas with unmet needs.

### Supplementary Information

**Additional file 1.**

**Additional file 2.**

## Data Availability

The datasets used and/or analyzed during the current study are available from the corresponding author on reasonable request.

## Abbreviations

HZ: Herpes zoster
PHN: Postherpetic neuralgia
QALY: Quality-adjusted life years
STIKO: Ständige Impfkommission (Standing Committee on Vaccination)
UV: Ultraviolet
VZV: Varicella zoster virus
