# Supplementary material for: Evaluating public interest in herpes zoster in Germany by leveraging the internet: a retrospective search data analysis
Source: BMC Public Health. 2023 Aug 15;23:1546. doi: 10.1186/s12889-023-16463-4 (PMC10426197; doi:10.1186/s12889-023-16463-4)
Supplement: Supplementary file 1 — Additional file 1. [file 12889_2023_16463_MOESM1_ESM.docx]

Supplementary Table 1: Average monthly herpes zoster-related web search volume per 100,000 inhabitants for Germany as a whole and its sixteen federal states from October 2016 to September 2020 in descending order

| State | Average monthly search volume per 100,000 inhabitants | Standard deviation |
| --- | --- | --- |
| Hamburg | 963.5 | 211.7 |
| Saarland | 855.7 | 188.7 |
| Bremen | 850.1 | 147.0 |
| Berlin | 728.4 | 136.7 |
| Mecklenburg-Western Pomerania | 725.2 | 175.4 |
| Thuringia | 683.3 | 146.4 |
| Saxony-Anhalt | 679.0 | 157.4 |
| Schleswig-Holstein | 666.2 | 169.6 |
| Saxony | 655.3 | 155.4 |
| Brandenburg | 647.1 | 164.0 |
| Hesse | 635.7 | 143.3 |
| Rhineland-Palatinate | 627.0 | 145.2 |
| Lower Saxony | 585.2 | 141.6 |
| North Rhine-Westphalia | 581.2 | 152.8 |
| Bavaria | 546.9 | 144.1 |
| Baden-Württemberg | 541.9 | 138.3 |
| Germany | 521.5 | 142 |
