# Supplementary material for: Evaluating public interest in herpes zoster in Germany by leveraging the internet: a retrospective search data analysis
Source: BMC Public Health. 2023 Aug 15;23:1546. doi: 10.1186/s12889-023-16463-4 (PMC10426197; doi:10.1186/s12889-023-16463-4)
Supplement: Supplementary file 2 — Additional file 2. [file 12889_2023_16463_MOESM2_ESM.pdf]

Supplementary Table 2: List of relevant keywords and their respective categories generated for the web search terms "herpes zoster" and "Gürtelrose" (shingles) in Germany

Keyword	Translation	Categories
zoster	zoster	General
gürtelrose	shingles	General
gürtelrose ansteckend	shingles contagious	Contagiousness
gürtelrose symptome	shingles symptoms	Symptoms
gesichtsrose	facial shingles	Localization
gürtelrose im gesicht	shingles in face	Localization
gürtelrose behandlung	shingles treatment	Therapy
gürtelrose dauer	shingles duration	General
ist gürtelrose ansteckend	is shingles contagious	Contagiousness
gürtelrose gesicht	shingles face	Localization
zoster sine herpete	zoster sine herpete	Symptoms
zoster ophthalmicus	zoster ophthalmicus	Localization
gürtelrose behandlung hausmittel	shingles treatment home remedy	Therapy
gürtelrose schmerzen	shingles pain	Symptoms
varizella zoster	varicella zoster	Causes
symptome gürtelrose	symptoms shingles	Symptoms
gürtelrose verlauf	shingles progression	General
gürtelrose am kopf	shingles on the head	Localization
gürtelrose schwangerschaft	shingles during pregnancy	Demographics
gürtelrose ursachen	shingles causes	Causes
post zoster neuralgie	post-zoster neuralgia	Complications
herpes zoster therapie	herpes zoster therapy	Therapy
ansteckung gürtelrose	contagion of shingles	Contagiousness
gürtelrose ohne ausschlag	shingles without rash	Symptoms
gürtelrose selbsttest	shingles self-test	Information
gürtelrose anfangsstadium	early stage of shingles	General
gürtelrose am hals	shingles on the neck	Localization
herpes zoster gesicht	facial herpes zoster	Localization
gürtelrose ansteckungsgefahr	contagiousness of shingles	Contagiousness
gürtelrose rücken	shingles on the back	Localization
gürtelrose am auge	shingles on the eye	Localization
gürtelrose auge	eye shingles	Localization
gürtelrose wikipedia	shingles wikipedia	Information
kopfrose	head shingles	Localization
gürtelrose am bein	shingles on the leg	Localization
gürtelrose bauch	shingles on the abdomen	Localization

gürtelrose bei kindern	shingles in children	Demographics
gürtelrose bein	leg shingles	Localization
behandlung gürtelrose	treatment shingles	Therapy
gürtelrose hals	shingles neck	Localization
gürtelrose am arm	shingles on arm	Localization
gürtelrose kind	shingles child	Demographics
gürtelrose ohne schmerzen	shingles without pain	Symptoms
windpocken gürtelrose	chickenpox shingles	Causes
postherpetische neuralgie	postherpetic neuralgia	Complications
gürtelrose ursache psychisch	shingles psychological causes	Causes
gürtelrose kopf	shingles head	Localization
herpes zoster auge	herpes zoster eye	Localization
herpes zoster ansteckung	herpes zoster contagion	Contagiousness
gürtelrose windpocken	shingles chicken pox	Causes
ophthalmicus	ophthalmicus	Localization
zoster neuralgie	zoster neuralgia	Complications
herpes zoster symptome	herpes zoster symptoms	Symptoms
nervenschmerzen nach gürtelrose	nerve pain after shingles	Complications
gürtelrose arm	shingles on the arm	Localization
gürtelrose baby	shingles in babies	Demographics
gesichtsrose ansteckend	facial shingles contagious	Localization, Contagiousness
gürtelrose ohne bläschen	shingles without blisters	Symptoms
gürtelrose brust	shingles on the chest	Localization
gürtelrose anfang	early signs of shingles	General
gürtelrose kopfhaut	shingles on the scalp	Localization
gürtelrose therapie	shingles treatment	Therapy
gürtelrose tödlich	fatal shingles	Complications
rosengürtel	shingles	General
gürtelrose in der schwangerschaft	shingles in pregnancy	Demographics
gürtelrose gefährlich	shingles dangerous	Complications
gürtelrose übertragung	transmission of shingles	Contagiousness
gürtelrose ausschlag	shingles rash	Symptoms
gürtelrose am fuß	shingles on the foot	Localization
gürtelrose hausmittel	home remedies for shingles	Therapy
gürtelrose unter der brust	shingles under the breast	Localization
gürtelrose im mund	shingles in the mouth	Localization
gürtelrose hand	shingles on the hand	Localization
gürtelrose rückfall symptome	shingles recurrence symptoms	Information, Symptoms

gürtelrose oberschenkel	shingles on the thigh	Localization
gesichtsrose hausmittel	facial shingles home remedies	Localization, Therapy
postzosterische neuralgie	postherpetic neuralgia	Complications
gürtelrose meldepflichtig	shingles reportable	Information
gürtelrose ansteckend baby	contagious shingles in babies	Contagiousness, Demographics
gürtelrose ansteckend kleinkind	contagious shingles in toddlers	Contagiousness, Demographics
gürtelrose im gesicht behandlung	treatment of shingles on the face	Localization, Therapy
herpes im ohr	herpes in the ear	Localization
herpes am rücken	herpes on the back	Localization
gürtelrose im ohr	shingles in the ear	Localization
gürtelrose langzeitfolgen	long-term effects of shingles	Complications
gürtelrose mehrmals	shingles multiple times	Information
gürtelrose kleinkind	shingles in toddlers	Demographics
gürtelrose im auge	shingles in the eye	Localization
gürtelrose am bauch	shingles on the abdomen	Localization
gürtelrose schmerzen nach jahren	shingles pain after years	Symptoms, Complications
ursache gürtelrose	shingles cause	Causes
gürtelrose frühstadium	early stage of shingles	General
gürtelrose schwanger	shingles in pregnancy	Demographics
gürtelrose schmerzen im rücken	shingles pain in the back	Symptoms, Localization
ansteckungsgefahr gürtelrose	risk of contagion for shingles	Contagiousness
gürtelrose stress	shingles stress	Causes
gürtelrose im gesicht ansteckend	shingles in face contagious	Localization, Contagiousness
gürtelrose nervenschmerzen therapie	shingles nerve pain therapy	Complications, Symptoms, Therapy
gürtelrose ohr	shingles ear	Localization
gesichtsgürtelrose	shingles in the face	Localization
gürtelrose am po	shingles on buttocks	Localization
gürtelrose herpes	shingles herpes	Other Diseases
herpes am bein	herpes on leg	Localization
gürtelrose schulter	shingles shoulder	Localization
gürtelrose fuß	shingles foot	Localization
erschöpfungszustand nach gürtelrose	exhaustion after shingles	Complications
gürtelrose auslöser	shingles triggers	Causes
schmerzen bei gürtelrose	pain with shingles	Symptoms

herpes zoster doccheck	herpes zoster doccheck	Information
gürtelrose krankheit	shingles disease	General
gürtelrose behandlungsdauer	duration of shingles treatment	Therapy
herpes am ohr	herpes on the ear	Localization
gürtelrose am oberschenkel	shingles on the thigh	Localization
gürtelrose am rücken	shingles on the back	Localization
gürtelrose nacken	shingles on the neck	Localization
gürtelrose nicht krankgeschrieben	shingles without sick leave	Sick Leave
zoster generalisatus	zoster generalisatus	Localization
gürtelrose bläschen	shingles blisters	Symptoms
windpocken herpes	chickenpox herpes	Causes
ist eine gürtelrose ansteckend	is shingles contagious	Contagiousness
dauer gürtelrose	duration of shingles	General
gürtelrose nervenschmerzen	shingles nerve pain	Symptoms
gürtelrose folgen	shingles consequences	Complications
schmerzmittel bei gürtelrose	pain medication for shingles	Therapy, Symptoms
gürtelrose durch stress	shingles caused by stress	Causes
gürtelrose an der hand	shingles on the hand	Localization
gürtelrose diagnose	shingles diagnosis	General
gürtelrose achsel	shingles in the armpit	Localization
zoster therapie	zoster therapy	Therapy
gürtelrose rückenschmerzen	shingles back pain	Localization, Symptoms
gürtelrose heilung	shingles cure	Therapy
gürtelrose dauer ansteckungsgefahr	duration of shingles contagion risk	Contagiousness
herpes zoster schwangerschaft	herpes zoster in pregnancy	Demographics
schmerzen gürtelrose	shingles pain	Symptoms
herpes rücken	herpes on the back	Localization
anfangsstadium gürtelrose	early stage of shingles	General
herpes sine herpete	herpes sine herpete	Symptoms
gürtelrose doccheck	shingles doccheck	Information
gürtelrose am ohr	shingles on the ear	Localization
post zoster neuralgie naturheilkunde	post-herpetic neuralgianaturopathy	Complications, Therapy
juckt gürtelrose	does shingles itch	Symptoms
herpes ohr	herpes on the ear	Localization
gürtelrose mund	shingles in the mouth	Localization
gürtelrose stirn	shingles on the forehead	Localization
auslöser gürtelrose	shingles triggers	Causes

gesichtsrose symptome	facial shingles symptoms	Localization, Symptoms
leichte gürtelrose	mild shingles	Symptoms
gürtelrose sonne	shingles sun	Causes
hausmittel gegen gürtelrose	home remedies for shingles	Therapy
herpes gürtelrose	herpes shingles	General
herpes zoster infektion	herpes zoster infection	General
beginnende gürtelrose	early stage of shingles	General
schmerzen nach gürtelrose	pain after shingles	Complications
herpes am bauch	herpes on the abdomen	Localization
gürtelrose spanisch	shingles spanish	General
gürtelrose nase	shingles nose	Localization
rosengürtel krankheit	shingles disease	General
therapie gürtelrose	shingles therapy/treatment	Therapy
verlauf gürtelrose	progression shingles	General
gürtelrose krankenhaus	shingles hospital	Therapy
herpes zoster verlauf	herpes zoster progression	General
gürtelrose schmerzen ohne ausschlag	shingles pain without a rash	Symptoms
gürtelrose unbehandelt	untreated shingles	Therapy
gürtelrose erreger	shingles-causing pathogen	Causes
gesichtsrose behandlung	facial shingles treatment	Localization, Therapy
herpes zoster sine herpete	herpes zoster sine herpete	Symptoms
herpes zoster neuralgie	herpes zoster neuralgia	Symptoms, Complications
gürtelrose im kopf	shingles in the head	Localization
gürtelrose schmerzmittel	shingles pain medication	Therapy, Symptoms
ist gürtelrose ansteckend für erwachsene	is shingles contagious for adults	Contagiousness, Demographics
innere gürtelrose	internal shingles	Localization
krankheit gürtelrose	disease shingles	General
zoster neuralgie schmerztherapie	zoster neuralgia pain therapy	Complications, Therapy, Symptoms
gűrtelrose	shingles	General
gesichtsrose dauer	duration of facial shingles	Localization
herpes am kopf	herpes on the head	Localization
ausschlag gürtelrose	shingles rash	Symptoms
gürtelrose ohne windpocken	shingles without chickenpox (note: shingles can occur without a history of chickenpox)	Causes
ist die gürtelrose ansteckend	is shingles contagious	Contagiousness

gürtelrose nervenschmerzen hausmittel	home remedies for shingles nerve pain	Complications, Symptoms, Therapy
gürtelrose kniekehle	shingles in the back of the knee	Localization
gürtelrose ansteckungszeit	shingles incubation period	Contagiousness
gürtelrose finger	shingles finger	Localization
kann man gürtelrose mehrmals bekommen	can you get shingles multiple times	Information
übertragung gürtelrose	transmission of shingles	Contagiousness
windpocken und gürtelrose	chickenpox and shingles	Causes
herpes zoster im gesicht	herpes zoster on the face	Localization
herpes zoster arm	herpes zoster arm	Localization
gürtelrose an den beinen	shingles on the legs	Localization
ansteckungsgefahr bei gürtelrose	contagion risk for shingles	Contagiousness
gürtelrose handgelenk	shingles wrist	Localization
behandlung von gürtelrose	treatment of shingles	Therapy
gürtelros	shingles	General
ist gürtelrose gefährlich	is shingles dangerous	Complications
gürtelrose po	shingles buttocks	Localization
herpes ausschlag gesicht	herpes rash on the face	Localization, Symptoms
gürtelrose narben	shingles scars	Complications
gürtelrose mehrmals hintereinander	recurring shingles	Information
gürtelrose ansteckend für schwangere	contagion risk of shingles for pregnant women	Contagiousness, Demographics
gürtelrose hiv	shingles hiv	Other Diseases
zoster gesicht	zoster on the face	Localization
post zoster neuralgie therapie	post-herpetic neuralgia therapy (treatment for nerve pain after shingles)	Therapy, Complications
gürtelrose am finger	shingles on the finger	Localization
kopfrose tödlich	shingles on the head deadly (note: "kopfrose tödlich" is not a standard medical term)	Localization, Complications
hautpflege nach gürtelrose	skin care after shingles	Therapy
gürtelrose auf dem kopf	shingles on the head	Localization
hausmittel gürtelrose	home remedies shingles	Therapy
herpes windpocken	herpes chickenpox	Causes
herpes zoster v1	herpes zoster v1	Localization
gürtelrose an der brust	shingles on the chest	Localization
gürtelrose krebs	shingles cancer	Other Diseases
gürtelrose fieber	shingles fever	Symptoms

gürtelrose psyche	shingles psyche	Causes
herpes zoster ohr	herpes zoster ear	Localization
gürtelrose hüfte	shingles hip	Localization
gürtelrose spätfolgen	shingles long-term effects	Complications
gürtelrose ansteckend für kinder	shingles contagious for children	Contagiousness, Demographics
gürtelrose krankheitsverlauf	shingles disease progression	General
gesichtsrose anfangsstadium	early stage facial shingles	Localization
gürtelrose bauchnabel	shingles navel	Localization
ansteckung bei gürtelrose	contagion with shingles	Contagiousness
herpes zoster gürtelrose	herpes zoster shingles	General
gürtelrose alkohol	shingles alcohol	Causes
herpes zoster mund	herpes zoster in the mouth	Localization
gürtelrose erwachsene	shingles adults	Demographics
gürtelrose trotz windpocken	shingles despite chickenpox	Causes
symptome bei gürtelrose	symptoms of shingles	Symptoms
gürtelrose auf spanisch	shingles in spanish	General
gürtelrose leiste	shingles groin	Localization
chronische gürtelrose	chronic shingles	Complications
gürtelrose ansteckung kinder	contagion of shingles in children	Contagiousness, Demographics
gürtelrose unter den achseln	shingles under the armpits	Localization
herpes zoster hand	herpes zoster on the hand	Localization
gürtelrose herpes zoster	shingles herpes zoster	General
kopfgürtelrose	shingles head	Localization
kann man an gürtelrose sterben	can shingles be fatal	Complications
zona herpes zoster	zona herpes zoster	General
ist gürtelrose tödlich	is shingles deadly	Complications
symptome gürtelrose bauch	symptoms of shingles abdomen	Symptoms, Localization
gürtelrose im gesicht dauer	duration of facial shingles	Localization
gürtelrose lippe	shingles lip	Localization
herpes zoster baby	herpes zoster baby	Demographics
gürtelrose ohne hautausschlag	shingles without skin rash	Symptoms
gürtelrose woher	shingles origin	Causes
kann man gürtelrose mehrfach bekommen	can you get shingles multiple times	Information
spätfolgen gürtelrose	long-term effects of shingles	Complications
gürtelrose heilungsdauer	duration of shingles healing	General
gürtelrose natürlich behandeln	natural treatment for shingles	Therapy
zinksalbe bei gürtelrose	zinc ointment for shingles	Therapy

stiko gürtelrose	stiko shingles	Information, Vaccine
gürtelrose im nacken	shingles on nape	Localization
gürtelrose verlauf dauer	shingles course duration	General
herpes zoster kopf	herpes zoster head	Localization
ansteckungszeit gürtelrose	contagion period for shingles	Contagiousness
erreger gürtelrose	shingles-causing pathogen	Causes
gürtelrose im genitalbereich	shingles in the genital area	Localization
ist herpes zoster ansteckend	is herpes zoster contagious?	Contagiousness
schutzimpfung gürtelrose	shingles vaccination	Vaccine
herpes zoster kopfhaut	herpes zoster scalp	Localization
gürtelrose rückfall	shingles recurrence	Information
ist gürtelrose meldepflichtig	is shingles reportable	Information
gürtelrose chronisch	chronic shingles	Complications
zoster ophthalmicus therapie	zoster ophthalmicus therapy	Localization, Therapy
gürtelrose in schwangerschaft	shingles in pregnancy	Demographics
symptome einer gürtelrose	symptoms of shingles	Symptoms
post zoster neuralgie kopf	post-herpetic neuralgia head	Complications, Localization
kopfrose symptome	symptoms shingles on the head	Localization, Symptoms
gürtelrose krankheitsdauer	duration of shingles disease	General
gürtelrose intimbereich	shingles genital area	Localization
gürtelrose mit 30	shingles at the age of 30	Demographics
herpes bein	herpes on the leg	Localization
unsichtbare gürtelrose	invisible shingles	Symptoms
post zoster	post zoster	Complications
symptome gürtelrose rücken	symptoms shingles back	Symptoms, Localization
gesichtsrose auge	facial shingles eye	Localization
gürtelrose am knie	shingles on the knee	Localization
gürtelrose bei erwachsenen	shingles in adults	Demographics
gürtelrose und sonne	shingles and sun	Causes
gürtelrose juckt	shingles itches	Symptoms
nervenschmerzen gürtelrose	nerve pain shingles	Symptoms, Complications
gürtelrose unterarm	shingles forearm	Localization
zoster ansteckend	zoster congaious	Contagiousness
gürtelrose mehrfach	recurring shingles	Information
zoster infektion	zoster infection	General
zoster auge	zoster eye	Localization
gürtelrose sterben	shingles death	Complications

symptome herpes zoster	symptoms herpes zoster	Symptoms
gürtelrose am handgelenk	shingles on the wrist	Localization
gürtelrose gesicht dauer	duration facial shingles	Localization
herpes bauch	herpes abdomen	Localization
gürtelrose ellenbogen	shingles elbow	Localization
behandlung bei gürtelrose	treatment for shingles	Therapy
herpes zoster schmerzen	herpes zoster pain	Symptoms
gürtelrose in der nase	shingles in the nose	Localization
gürtelrose alternativ behandeln	alternative treatment for shingles	Therapy
gürtelrose arbeitsunfähig	shingles work incapacity	Sick Leave
herpes zoster übertragung	herpes zoster transmission	Contagiousness
gürtelrose dauer der erkrankung	duration of shingles disease	General
gürtelrose blutwerte	shingles blood values	Information
gürtelrose und schwangerschaft	shingles during pregnancy	General
gürtelrose erkennungszeichen	shingles signs	Symptoms
herpes auf der stirn	herpes on the forehead	Localization
gürtelrose oberarm	shingles on the upper arm	Localization
masern gürtelrose	measles shingles	Other Diseases
gesichtsrose kopfhaut	facial shingles scalp	Localization
gürtelrose zinksalbe	shingles zink ointment	Therapy
herpes zoster kind	herpes zoster child	Demographics
gürtelrose und windpocken	shingles and chickenpox	Causes
gürtelrose ansteckung schwangerschaft	contagion of shingles in pregnancy	Contagiousness, Demographics
gürtelrose arbeitsunfähig wie lange	work incapacity duration for shingles	Sick Leave
herpes nervenschmerzen	herpes nerve pain	Symptoms, Complications
hausmittel bei gürtelrose	home remedies for shingles	Therapy
gürtelrose im alter	shingles in old age	Demographics
gürtelrose im intimbereich	shingles in the genital area	Localization
gürtelrose erste symptome	initial symptoms of shingles	Symptoms
virostatika gürtelrose	antivirals shingles	Therapy
gürtelrose durch grippeimpfung	shingles caused by flu vaccination	Causes
gürtelrose gesicht behandlung	facial shingles treatment	Localization, Therapy
diagnose gürtelrose	diagnosis shingles	General
gürtelrose ansteckend oder nicht	shingles contagious or not	Contagiousness
wikipedia gürtelrose	wikipedia shingles	Information
innere gürtelrose ohne ausschlag symptome	internal shingles without rash symptoms	Localization, Symptoms
herpes zoster rücken	herpes zoster back	Localization

therapie bei gürtelrose	treatment for shingles	Therapy
nervenschmerzen bei gürtelrose	nerve pain with shingles	Symptoms, Complications
gürtelrose bei schwangeren	shingles in pregnant women	Demographics
gürtelrose masern	shingles measles	Other Diseases
gürtelrose im mund ansteckend	contagiousness of shingles in mouth	Localization, Contagiousness
gürtelrose krankenschein	shingles medical certificate	Sick Leave
gürtelrose in der frühen schwangerschaft	shingles in early pregnancy	Demographics
gürtelrose am ganzen körper	shingles all over the body	Localization
ursachen für gürtelrose	causes of shingles	Causes
anfang gürtelrose	early stage shingles	General
gürtelrose anfangsstadium gesicht	initial symptoms of facial shingles	Localization
schwangerschaft gürtelrose	shingles during pregnancy	Demographics
rki gürtelrose	rki shingles	Information
gürtelrose am mund	shingles in mouth	Localization
gürtelrose test	shingles test	Information
gürtelrose hausmittel schmerzen	shingles home remedy pain	Therapy, Symptoms
neuralgie nach zoster	neuralgia after zoster	Complications
schmerzmittel gürtelrose	pain remedy shingles	Therapy, Symptoms
gürtelrose knie	shingles knee	Localization
gürtelrose genitalbereich	shingles genital area	Localization
gürtelrose säugling	shingles infant	Demographics
gürtelrose wundrose	shingles cellulitis	Other Diseases
gürtelrose bluttest	shingles blood test	Information
gürtelrose am auge ansteckend	shingles in eye contagious	Localization, Contagiousness
herpes zoster ohne bläschen	herpes zoster without blisters	Symptoms
ansteckung herpes zoster	contagiousness herpes zoster	Contagiousness
zoster sine herpete wikipedia	zoster sine herpete wikipedia	Information, Symptoms
herpes zoster im mund	herpes zoster in mouth	Localization
gürtelrose im ohr gesichtslähmung	shingles in ear facial paralysis	Localization, Symptoms
herpes zoster thoracalis	herpes zoster thoracalis	Localization
gürtelrose bei jungen menschen	shingles in young people	Demographics
herpes zoster bein	herpes zoster leg	Localization
herpes zoster ohne ausschlag	herpes zoster without rash	Symptoms
gesichtsgürtelrose ursache	facial shingles causes	Localization, Causes

sta je herpes zoster	sta je herpes zoster	General
gürtelrose taubheitsgefühl	shingles numbness	Symptoms
post zoster neuralgie dauer	post zoster neuralgia duration	Complications
gürtelrose bei babys	shingles in babies	Demographics
therapie bei gürtelrose	treatment after shingles	Therapy
gürtelrose zunge	shingles tongue	Localization
kopfrose ansteckend	contagious head shingles	Localization, Contagiousness
gürtelrose naturheilmittel	natural remedies shingles	Therapy
robert franz gürtelrose	robert franz shingles	Therapy
zoster im gesicht	zoster in the face	Localization
impfungen gegen gürtelrose	vaccinations against shingles	Vaccine
gürtelrose krankmeldung	sick leave for shingles	Sick Leave
herpes zoster in der schwangerschaft	herpes zoster in pregnancy	Demographics
symptome gürtelrose gesicht	symptoms shingles face	Symptoms, Localization
gürtelrose infektion	shingles infection	General
rosa gürtel krankheit	shingles disease	General
gürtelrose ohne behandlung	shingles without treatment	Therapy
gürtelrose am kopf symptome	symptoms of shingles on the head	Localization, Symptoms
gürtelrose gesicht ansteckend	contagious shingles in the face	Localization, Contagiousness
gürtelrose unterm arm	shingles under the arm	Localization
varizella zoster therapie	varicella zoster therapy	Therapy
welche schmerzmittel bei gürtelrose	recommended painkillers for shingles	Therapy, Symptoms
gürtelrose abheilung	healing process of shingles	General
erste symptome gürtelrose	early symptoms of shingles	Symptoms
herpes zoster rki	herpes zoster rki	Information
krankheitsverlauf gürtelrose	disease progression of shingles	General
herpes an der brust	herpes chest	Localization
sine herpete	sine herpete	Symptoms
gürtelrose an den händen	shingles on the hands	Localization
gürtelrose fußsohle	shingles on the sole of the foot	Localization
gürtelrose schmerzen dauer	shingles pain duration	Symptoms
herpes zoster am auge	herpes zoster on eye	Localization
herpes zoster hals	herpes zoster neck	Localization
rosen gürtel	shingles	General
gürtelrose gesicht symptome	shingles face symptoms	Localization, Symptoms

kann man gürtelrose bekommen wenn man windpocken hatte	can you get shingles when you had chickenpox	Causes
innere gürtelrose ohne ausschlag	internal shingles without rash	Localization, Symptoms
gürtelrose beidseitig	shingles both sides	Localization
gürtelrose dauer schmerzen	shingles duration pain	Symptoms
gürtelrose halsschmerzen	shingles throat pain	Localization, Symptoms
gürtelrose krankschreiben	shingles sick leave	Information
gürtelrose leichte form	shingles mild form	Symptoms
zoster v1	zoster v1	Localization
gürtelrose penis	shingles penis	Localization
zoster behandlung	zoster treatment	Therapy
gürtelrose bei kleinkindern	shingles in toddlers	Demographics
zoster ohne komplikation	zoster without complications	Complications
gürtelrose symptome frühstadium	shingles symptoms early stage	Symptoms
gürtelrose brust frau	shingles breast woman	Localization
gürtelrose ähnliche krankheiten	shingles similar diseases	Other Diseases
impfungen gürtelrose	vaccines shingles	Vaccine
windpockenimpfung gürtelrose	chickenpox vaccine shingles	Vaccine, Causes
diagnose gürtelrose ohne ausschlag	diagnosis shingles without rash	Symptoms
anfangsstadium gürtelrose symptome	early stage shingles symptoms	Symptoms
gesichtsrose verlauf	facial shingles progression	Localization
herpes zoster nase	herpes zoster nose	Localization
gesicht gürtelrose	face shingles	Localization
gürtelrose intimbereich behandlung	shingles genital area treatment	Localization, Therapy
gürtelrose hinter dem ohr	shingles behind the ear	Localization
gürtelrose symptome anfang	shingles symptoms beginning	Symptoms
gürtelrose am kopf ansteckend	shingles on the head contagious	Localization, Contagiousness
zoster schmerzen	zoster pain	Symptoms
auslöser für gürtelrose	cause for shingles	Causes
gürtelrose am kinn	shingles on chin	Localization
herpes zoster deutsch	herpes zoster german	General
herpes zoster kinder	herpes zoster children	Demographics
gürtelrose ohne schmerzen und fieber	shingles without pain and fever	Symptoms
gürtelrose am penis	shingles on penis	Localization
augen gürtelrose	eyes shingles	Localization
gürtelrose am hinterkopf	shingles on back of head	Localization

gürtelrose zoster	shingles zoster	General
ist gesichtsrose ansteckend	is facial shingles contagious	Contagiousness, Localization
post zoster neuralgie behandlung	post zoster neuralgia treatment	Complications, Therapy
gürtelrose nach windpocken	shingles after chickenpox	Causes
gürtelrose kommt wieder	shingles recurrence	Information
gürtelrose am rücken symptome	shingles on back symptoms	Localization, Symptoms
gürtelrose schwindelgefühle	shingles dizziness	Symptoms
rückenschmerzen gürtelrose	back pain shingles	Symptoms, Localization
gürtelrose keine schmerzen	shingles without pain	Symptoms
gürtelrose folgeschäden	shingles complications	Complications
zinksalbe gürtelrose	zinc cream for shingles	Therapy
zona zoster magyarul	zona zoster magyarul	General
gürtelrose auf der kopfhaut	shingles on the scalp	Localization
gürtelrose bauchschmerzen	shingles abdominal pain	Localization, Symptoms
gürtelrose gewichtsverlust	shingles weight loss	Symptoms
ernährungs docs gürtelrose	nutrition docs shingles	Information
gürtelrose fachbegriff	shingles medical term	General
gürtelrose nicht ansteckend	shingles not contagious	Contagiousness
gürtelrose am oberarm	shingles on upper arm	Localization
gürtelrose ist das ansteckend	shingles is that contagious	Contagiousness
herpes zoster dauer	herpes zoster duration	General
gürtelrose selbst behandeln	shingles self treatment	Therapy
gürtelrose am nacken	shingles on nape	Localization
herpes kopf	herpes head	Localization
versteckte gürtelrose	hidden shingles	Symptoms
gürtelrose erneutes auftreten	shingles recurrence	Information
apotheken umschau gürtelrose	apotheken umschau shingles	Information
gürtelrose durch windpocken	shingles because of chickenpox	Causes
gürtelrose bettruhe	shingles bed rest	Therapy
innerliche gürtelrose	internal shingles	Localization
unbehandelte gürtelrose	untreated shingles	Therapy
ist eine gürtelrose gefährlich	is shingles dangerous	Complications
windpocken herpes zoster	chickenpox herpes zoster	Causes
herpes zoster ursache	herpes zoster causes	Causes
schmerztherapie bei gürtelrose	pain therapy for shingles	Therapy, Symptoms
gürtelrose zentrum der gesundheit	shingles center for health	Information

gürtelrose am after	shingles on anus	Localization
gürtelrose oberkörper	shingles upper body	Localization
krankheit rosengürtel	disease shingles	General
gürtelrose hirnhautentzündung	shingles meningitis	Complications
hautausschlag gürtelrose	rash shingles	Symptoms
herpes zoster v2	herpes zoster v2	Localization
morbus zoster	morbus zoster	General
herpes zoster viren	herpes zoster viruses	Causes
gürtelrose apotheken umschau	shingles apotheken umschau	Information
post zoster neuralgie symptome	postherpetic neuralgia symptoms	Complications
kann man von gürtelrose sterben	can one die from shingles	Complications
herpes zoster generalisatus	herpes zoster generalisatus	Localization
zoster symptome	zoster symptoms	Symptoms
gürtelrose dauer der schmerzen	shingles duration of pain	Symptoms
herpes zoster cerebral	herpes zoster cerebral	Localization
herpes zoster bei kindern	herpes zoster in children	Demographics
gürtelrose in der ss	shingles in pregnancy	Demographics
gürtelrose symptome ohne ausschlag	shingles symptoms without rash	Symptoms
gürtelrose hautpflege	shingles skincare	Therapy
gürtelrose alter	shingles age	Demographics
ständig wiederkehrende gürtelrose	recurrent shingles	Information
gürtelrose after	shingles anus	Localization
gürtelrose psychisch	shingles psychological	Causes
gürtelrose erneuter ausbruch	shingles recurrence	Information
baby gürtelrose	baby shingles	Demographics
gürtelrose herz	shingles heart	Localization
herpes zoster meldepflichtig	herpes zoster reportable	Information
gürtelrose schwangerschaft therapie	shingles pregnancy therapy	Demographics, Therapy
gürtelrose wade	shingles calf	Localization
symptome von gürtelrose	symptoms shingles	Symptoms
gürtelrose geschlossen	shingles closed	General
gürtelrose im hals	shingles in throat	Localization
gürtelrose bei älteren menschen	shingles in older people	Demographics
gürtelrose an der schulter	shingles on the shoulder	Localization
gürtelrose ohrenschmerzen	shingles ear pain	Localization, Symptoms
gürtelrose ansteckend für babys	shingles contagious for babies	Contagiousness, Demographics
gürtelrose schutzimpfung	shingles vaccination	Vaccine

gürtelrose	shingles	General
gürtelrose augenlid	shingles eyelid	Localization
herpes zoster windpocken	herpes zoster chickenpox	Causes
gürtelrose mit 20	shingles at 20	Demographics
gürtelrose armbeuge	shingles elbow crease	Localization
gürtelrose symptome und behandlung	shingles symptoms and treatment	Symptoms, Therapy
symptome gesichtsrose	symptoms facial shingles	Symptoms, Localization
gürtelrose in der leistengegend	shingles in the groin area	Localization
die gürtelrose	shingles	General
gürtelrose nackenschmerzen	shingles neck pain	Localization, Symptoms
windpockenvirus	varicella-zoster virus	Causes
lyranda bei gürtelrose	lyranda for shingles	Therapy
wiederkehrende gürtelrose	recurrent shingles	Information
behandlung gürtelrose bei erwachsenen	treatment of shingles in adults	Therapy, Demographics
varizella zoster symptome	varicella-zoster symptoms	Symptoms
gürtelrose im gesicht symptome	shingles in face symptoms	Localization, Symptoms
gürtelrose scheide	shingles vagina	Localization
herpes zoster brust	herpes zoster chest	Localization
gürtelrose ursache stress	shingles cause stress	Causes
gürtel krankheit	shingles disease	General
gürtelrose hinterkopf	shingles back of head	Localization
herpes oder gürtelrose	herpes or shingles	Other Diseases
schutzimpfung gegen gürtelrose	vaccination against shingles	Vaccine
herpes auf dem rücken	herpes on the back	Localization
gesichtsrose nase	facial shingles nose	Localization
gürtelrose rki	shingles rki	Information
gürtelrose blutbild	shingles blood values	Information
dauer einer gürtelrose	duration of shingles	General
gürtelrose an der stirn	shingle on the forehead	Localization
postherpetische neuralgie behandlung	post herpetic neuralgia treatment	Therapy, Complications
symptome der gürtelrose	symptoms of shingles	Symptoms
therapie zoster	therapy zoster	Therapy
gürtelrose an den füßen	shingles on the feet	Localization
gürtelrose an der nase	shingles on the nose	Localization
gürtelrose hautarzt	shingles dermatologist	Therapy
gürtelrose spezialklinik	shingles special clinic	Therapy

rückenschmerzen bei gürtelrose	back pain from shingles	Symptoms, Localization
gürtelrose dauer ausschlag	shingles duration rash	Symptoms
gürtelrose oder herpes	shingles or herpes	Other Diseases
gürtelrose übertragbar	shingles transmissable	Contagiousness
kopf gürtelrose	head shingles	Localization
herpes zoster am kopf	herpes zoster on head	Localization
fieber bei gürtelrose	fever with shingles	Symptoms
folgen gürtelrose	consequences shingles	Complications
herpes neuralgie	herpes neuralgia	Symptoms, Complications
gürtelrose ansteckend wenn man windpocken hatte	shingles contagious when one had chickenpox	Causes, Contagiousness
gürtelrose beim kind	shingles in children	Demographics
verhaltensregel bei gürtelrose	behavioral rules for shingles	Information
gürtelrose geht nicht weg	shingles does not go away	Complications
gürtelrose im blut nachweisbar	shingles detectable in blood	Information
gürtelrose schmerztherapie	shingles pain treatment	Therapy, Symptoms
gürtelrose schienbein	shingles shin	Localization
zoster wikipedia	zoster wikipedia	Information
herpes zoster am fuß	herpes zoster on foot	Localization
gürtelrose unter der achsel	shingles under armpit	Localization
mit gürtelrose in die sonne	with shingles in the sun	Information
symptome gürtelrose brust	symptoms shingles chest	Symptoms, Localization
hautpflege bei gürtelrose	skin care with shingles	Therapy
gürtelrose ohne fieber	shingles without fever	Symptoms
herpes zoster grave	severe herpes zoster	Symptoms
herpes zoster anfangsstadium	herpes zoster early stage	General
zoster sine herpete dauer	zoster sine herpete duration	Symptoms
gürtelrose heilungsprozess	shingles healing process	General
gürtelrose dekoltee	shingles chest	Localization
gürtelrose kinn	shingles chin	Localization
zosterschmerzen	zoster pain	Symptoms
ist gürtelrose im gesicht ansteckend	is shingles in the face contagious	Contagiousness, Localization
folgen einer gürtelrose	consequences of shingles	Complications
gürtelrose immer wieder	shingles always comes back	Information
gürtelrose hinterm ohr	shingles behind ear	Localization
gürtelrose unterschenkel	shingles calves	Localization
immer wieder gürtelrose	shingles recurrence	Information
verdacht auf gürtelrose	suspicion of shingles	General

gürtelrose schnelltest	shingles rapid test	Information
arsenicum album gürtelrose	arsenicum album shingles	Therapy
gürtelrose in der leiste	shingles in the groin area	Localization
gürtelrose kommt immer wieder	shingles always comes back	Information
gürtelrose schulterblatt	shingles shoulder blade	Localization
herpes zoster oberschenkel	herpes zoster thigh	Localization
windpocken und herpes	chickenpox and herpes	Causes
herpes zoster blutwerte	herpes zoster lab values	Information
gürtelrose krankgeschrieben	shingles sick leave	Sick Leave
gürtelrose nebenwirkungen	shingles side effects	Symptoms
gürtelrose auge symptome	shingles eye symptoms	Localization, Symptoms
gürtelrose am unterschenkel	shingles on calves	Localization
gürtelrose starke schmerzen	shingles severe pain	Symptoms
gürtelrose auf der brust	shingles on chest	Localization
gürtelrose am fußgelenk	shingles on ankle	Localization
gürtelrose behandlung alternativ	shingle alternative treatment	Therapy
generalisierter herpes zoster	generalized herpes zoster	Localization
gürtelrose hautausschlag	shingles rash	Symptoms
gürtelrose nicht behandeln	shingles not treating	Therapy
gürtelrose steißbein	shingles coccyx	Localization
gürtelrose cortisonsalbe	shingles cortisone cream	Therapy
gürtelrose am gesäß	shingles on the buttocks	Localization
gürtelrose erfahrungsberichte	shingles experiences	Information
gürtelrose immun	shingles immune	General
varizella zoster infektion	varicella-zoster infection	General
gürtelrose brustkorb	shingles thorax	Localization
herpes zoster ohne schmerzen	herpes zoster without pain	Symptoms
herpes zoster im ohr	herpes zoster in the ear	Localization
zoster zona	zoster zona	General
gürtelrose baby symptome	shingles baby symptoms	Demographics, Symptoms
kinder gürtelrose	children shingles	Demographics
zoster schmerzen therapie	zoster pain therapy	Therapy, Symptoms
gürtelrose lebensgefährlich	shingles life threatening	Complications
herpes und gürtelrose	herpes and shingles	General
gürtelrose nervenkrankheit	shingles nerve disease	General
zentrum der gesundheit gürtelrose	center of health shingles	Information
zoster oticus symptome	zoster oticus symptoms	Localization, Symptoms

krankheitsdauer gürtelrose	disease duration shingles	General
windpocken durch gürtelrose	chickenpox caused by shingles	Causes
gürtelrose am ellenbogen	shingles elbow	Localization
gürtelrose am knöchel	shingles ankle	Localization
gürtelrose innen	shingles internal	Localization
herpes auf dem kopf	herpes on head	Localization
gürtelrose fussgelenk	shingles ankle	Localization
gürtelrose unter der haut	shingles under the skin	Localization
gürtelrose an mehreren stellen	shingles on many areas	Localization
gürtelrose dauer ansteckung	shingles duration contagiousness	Contagiousness
gürtelrose ssw	shingles pregnancy week	Demographics
alles über gürtelrose	everything about shingles	General
gürtelrose baby ansteckung	shingles baby contagiousness	Contagiousness, Demographics
gürtelrose depression	shingles depression	Other Diseases
gürtelrose gesäß	shingles buttocks	Localization
zoster schwangerschaft	zoster pregnancy	Demographics
herpes zoster ausschlag	herpes zoster rash	Symptoms
wundrose gürtelrose	cellulitis shingles	Other Diseases
gürtelrose an der lippe	shingles on lip	Localization
gürtelrose im anfangsstadium	shingles in early stage	General
gürtelrose leicht	shingles mild	Symptoms
gürtelrose am bein ansteckend	shingles on leg contagious	Localization, Contagiousness
gürtelrose differentialdiagnose	shingles differential diagnosis	Other Diseases
herpes zoster stirn	herpes zoster forehead	Localization
gürtelrose ausschlag dauer	shingles rash duration	Symptoms
schmerztherapie gürtelrose	pain therapy shingles	Therapy, Symptoms
zoster ophthalmicus ohne ausschlag	zoster ophthalmicus without rash	Localization, Symptoms
herpes zoster bauch	herpes zoster abdomen	Localization
test gürtelrose	test shingles	Information
zoster gürtelrose	zoster shingles	General
atypische gürtelrose	atypical shingles	General
kopfrose dauer	shingles on head duration	Localization
gürtelrose bei jugendlichen	shingles in young people	Demographics
ich habe gürtelrose kann ich mein baby anstecken	i have shingles can i infect my baby	Contagiousness, Demographics
zoster am auge	zoster in eye	Localization
gürtelrose gesichtslähmung	shingles facial paralysis	Symptoms, Localization

gürtelrose nach windpockenimpfung	shingles after chickenpox vaccine	Causes, Vaccine
gürtelrose ganzer körper	shingles entire body	Localization
gürtelrose heilpraktiker	shingles naturopath	Therapy
gürtelrose am schienbein	shingles on shin	Localization
wikipedia herpes zoster	wikipedia herpes zoster	Information
gürtelrose am unterarm	shingles on forearm	Localization
gürtelrose wiederholter ausbruch	shingles repeated outbreak	Information
herpes gesichtsnerv	shingles facial nerve	Localization
gesichtsrose ohne ausschlag	facial shingles without rash	Localization, Symptoms
nach gürtelrose	after shingles	General
gürtelrose einmalig	shingles one time	Information
herpes zoster im auge	herpes zoster in eye	Localization
gürtelrose natürlich heilen	shingle treat naturally	Therapy
gürtelrose entzündungswerte	shingles infectious values	Information
gürtelrose trotz windpockenimpfung	shingles despite chickenpox vaccine	Vaccine, Causes
ist gürtelrose ansteckbar	is shingles contagious	Contagiousness
gürtelrose herzrasen	shingles heart palpitations	Symptoms
gürtelrose symptome rücken	shingles symptoms back	Symptoms, Localization
symptome für gürtelrose	symptoms of shingles	Symptoms
darf man mit gürtelrose in die sonne	can you go with shingles in the sun	Information
gürtelrose neugeborenes	shingles newborn	Demographics
narben nach gürtelrose	scars after shingles	Complications
gürtelrose durch sonne	shingles because of the sun	Causes
gürtelrose beschwerden	shingles complaints	Symptoms
gürtelrose unter der brust frau	shingles under breast woman	Localization
mehrmals gürtelrose	shingles recurrence	Information
zoster sine herpete symptome	zoster sine herpete symptoms	Symptoms
mezereum gürtelrose	mezereum shingles	General
gürtelrose mann	shingles man	Demographics
gürtelrose nur ein bläschen	shingles only one blister	Symptoms
gürtelrose ursachen bei jungen menschen	shingles causes in young people	Causes, Demographics
zoster arm	zoster arm	Localization
gürtelrose am zeh	shingles on toe	Localization
kann man gürtelrose bekommen wenn man gegen windpocken geimpft ist	can you get shingles when you are vaccinated against chickenpox	Vaccine, Causes
gürtelrose pflaster	shingles bandage	Therapy

gürtelrose auslöser stress	shingles trigger stress	Causes
gürtelrose leichter verlauf	shingles mild disease course	Symptoms
gürtelrose schule	shingles school	Contagiousness
gürtelrose unbemerkt	shingles unnoticed	Symptoms
nebenwirkungen gürtelrose	side effects shingles	Complications
bettruhe bei gürtelrose	bed rest during shingles	Therapy
gürtel rosa krankheit	shingles disease	General
post herpes zoster neuralgie	poster herpetic neuralgia	Complications
gürtelrose brennende schmerzen	shingles burning pain	Symptoms
innere gürtelrose wikipedia	internal shingles wikipedia	Localization, Information
folgen von gürtelrose	consequences of shingles	Complications
gürtelrose brustbereich	shingles chest	Localization
gürtelrose lippenherpes	shingles lip herpes	Other Diseases
halsschmerzen nach gürtelrose	throat pain after shingles	Complications, Localization
rückfall gürtelrose	shingles recurrence	Information
gürtelrose achselbereich	shingles armpit	Localization
gürtelrose immunsystem	shingles immune system	General
gürtelrose zahnschmerzen	shingles tooth pain	Symptoms
gürtelrose nicht sichtbar	shingles invisible	Symptoms
gürtelrose zwischen der brust	shingles between breasts	Localization
stress gürtelrose	stress shingles	Causes
kopfrose behandlung	shingles on head treatment	Localization, Therapy
schmerzmittel gegen gürtelrose	pain medication for shingles	Therapy, Symptoms
gürtelrose muskelschmerzen	shingles muscle pain	Symptoms
herpes im ohr symptome	herpes in ear symptoms	Localization, Symptoms
gürtelrose unter brust	shingles under breast	Localization
gürtelrose brustschmerzen	shingles chest pain	Localization, Symptoms
ist gürtelrose gefährlich für schwangere	is shingles dangerous for pregnant women	Complications, Demographics
herpes auf der brust	herpes on the chest	Localization
herpes zoster fuß	herpes zoster foot	Localization
gürtelrose ansteckend schwanger	shingles contagious pregnant	Contagiousness, Demographics
gürtelrose am augenlid	shingles on eyelid	Localization
herpes zoster chronisch	herpes zoster chronic	Complications
gürtelrose leistengegend	shingles groin	Localization
gürtelrose erkältung	shingles cold	Other Diseases

gürtelrose schlechte blutwerte	shingles severe blood values	Information
gürtelrose ohne bläschen ansteckend	shingles without blisters contagious	Symptoms, Contagiousness
gürtelrose wie übertragbar	shingles how transmited	Contagiousness
herpes zoster am bein	herpes zoster on leg	Localization
kann man mehrmals gürtelrose bekommen	can you get shingles more than once	Information
gürtelrose auf der stirn	shingles on forehead	Localization
gürtelrose frauen	shingles women	Demographics
wird man bei gürtelrose krankgeschrieben	can you go on sick leave with shingles	Sick Leave
gürtelrose netdoktor	shingles netdoktor	Information
herpes zoster ansteckungsgefahr	shingles contagiousness	Contagiousness
innenliegende gürtelrose	internal shingles	Localization
meldepflicht gürtelrose	reportable shingles	Information
gürtelrose neuralgie	shingles neuralgia	Complications
herpes zoster bläschen	herpes zoster blisters	Symptoms
symptome gürtelrose bein	symptoms shingles leg	Symptoms, Localization
gürtelrose mit 40	shingles at 40	Demographics
herpes schulter	herpes shoulder	Localization
gesichtsrose ursache	facial shingles cause	Localization, Causes
gürtelrose leber	shingles liver	Localization
juckt eine gürtelrose	does shingles itch	Symptoms
post zoster schmerzen	poster zoster pain	Complications
zosterviren	zoster viruses	General
gürtelrose in der schwangerschaft behandlung	shingles during pregnancy treatment	Demographics, Therapy
gürtelrose und krebs	shingles and cancer	Other Diseases
gegen gürtelrose	against shingles	Therapy
gürtelrose auf dem rücken	shingles on the back	Localization
gürtelrose in jungen jahren	shingles in youth	Demographics
rücken gürtelrose	back shingles	Localization
ausschlag bei gürtelrose	rash shingles	Symptoms
herpes zoster lokaltherapie	herpes zoster local therapy	Therapy
gürtelrose ohne ausschlag symptome	shingles without rash symptoms	Symptoms
gürtelrose symptome gesicht	facial shingles symptoms face	Symptoms, Localization
alkohol bei gürtelrose	alcohol during shingles	Causes
gesichtsgürtelrose ansteckend	facial shingles contagious	Localization, Contagiousness

gürtelrose an der wade	shingles on calf	Localization
ist gürtelrose ansteckend für kinder	is shingles contagious for children	Contagiousness, Demographics
schafblattern gürtelrose	chickenpox shingles	Causes
gürtelrose unbehandelt folgen	untreated shingles consequences	Therapy, Complications
gürtelrose und herpes	shingles and herpes	Other Diseases
gürtelrose wirbelsäule	shingles spine	Localization
gürtelrose stellen	shingles localizations	Localization
gürtelrose hitze	shingles heat	Causes
gürtelrose schmerzen in der nacht	shingles pain at night	Symptoms
gürtelrose auf kopfhaut	shingles on scalp	Localization
gürtelrose infektionsweg	shingles transmission route	Contagiousness
übertragung herpes zoster	transmission shingles	Contagiousness
behandlung der gürtelrose	treatment of shingles in adults	Therapy
bläschen gürtelrose	blisters shingles	Symptoms
gürtelrose ohrmuschel	shingles pinna	Localization
herpes ausschlag körper	herpes rash body	Localization, Symptoms
schmerzmittel bei herpes zoster	pain medication for shingles	Therapy, Symptoms
darf man bei gürtelrose in die sonne	can you go with shingles in the sun	Information
erkältung nach gürtelrose	cold after shingles	Other Diseases
gesichtsrose therapie	facial shingles therapy	Localization, Therapy
gürtelrose alte menschen	shingles old people	Demographics
anfang einer gürtelrose	early stage shingles	General
gürtelrose jugendalter	shinges youth	Demographics
gürtelrose von innen	internal shingles	Localization
herpes zoster genitalbereich	herpes zoster genital area	Localization
ständige impfkommission gürtelrose	vaccination committee shingles	Information, Vaccine
gesichtszoster	facial shingles	Localization
gürtelrose und ms	shingles and ms	Other Diseases
gürtelrose gefährlich bei ms	shingles dangerous with ms	Other Diseases, Complications
gürtelrose hals anfangsstadium	shingles neck early stage	Localization
herpes zoster haut	herpes zoster skin	Symptoms
zovirax gürtelrose	zovirax shingles	Therapy
gürtelrose am hintern	shingles buttocks	Localization
gürtelrose stillzeit	shingles breastfeeding	Demographics
gürtelrose wange	shingles cheek	Localization

herpes und windpocken	herpes and chickenpox	Causes
ist gürtelrose ansteckend wenn man windpocken hatte	is shingles contagious when you had chickenpox	Contagiousness, Causes
gürtelrose knöchel	shingles ankle	Localization
gürtelrose rücken symptome	shingles back symptoms	Localization, Symptoms
gürtelrose in der kniekehle	shingles on back of knee	Localization
gürtelrose mit 80 jahren	shingles at 80	Demographics
herpes ausschlag am körper	herpes rash on body	Localization, Symptoms
herpes zoster hausmittel	herpes zoster home remedy	Therapy
geht gürtelrose von alleine weg	does shingles go away by itself	Therapy
gürtelrose naturheilkundlich behandeln	shingles naturopathic treatment	Therapy
gürtelrose spirituell	shingles spiritual	Causes
herpetes	herpetes	General
rückenschmerzen nach gürtelrose	back pain after shingles	Symptoms, Localization, Complications
zoster kopfhaut	zoster scalp	Localization
gesichtsrose stirn	facial shingles forehead	Localization
herpes an den beinen	herpes on legs	Localization
gürtelrose gefährlich für baby	shingles dangerous for baby	Complications, Demographics
gürtelrose ähnlich	shingles similar	Other Diseases
kleine gürtelrose	mild shingles	Demographics
bluttest gürtelrose	blood test shingles	Information
gürtelrose im blut nachweisen	shingles in blood detectable	Information
herpes unter der brust	herpes under breast	Localization
gürtelrose ansteckend ja oder nein	shingles contagious yes or no	Contagiousness
gürtelrose kinderkrankheit	shingles childhood disease	Demographics
heilungsdauer gürtelrose	healing duration shingles	General
rosengürtel ausschlag	shingles rash	Symptoms
gürtelrose bei kindern schule	shingles in children school	Demographics
herpes ohrmuschel	herpes on the ear	Localization
herpes zoster bluttest	herpes zoster blood test	Information
blutwerte bei gürtelrose	lab values for shingles	Information
gürtelrose im gesicht ursachen	shingles in face causes	Localization, Causes
symptome gürtelrose arm	symptoms shingles arm	Symptoms, Localization
gürtelrose mit 30 jahren	shingles at 30	Demographics
gürtelrose tröpfcheninfektion	shingles droplet transmission	Contagiousness

ms und gürtelrose	ms and shingles	Other Diseases
gürtelrose an den armen	shingles on the arms	Localization
gürtelrose oder ausschlag	shingles or rash	Other Diseases, Symptoms
narben gürtelrose	scars shingles	Complications
gürtelrose ansteckbar	shingles contagious	Contagiousness
gürtelrose hausarzt oder hautarzt	shingles gp or dermatologist	Therapy
herpes zoster intimbereich	herpes zoster genital area	Localization
mit gürtelrose ins krankenhaus	go to hospital with shingles	Therapy
gürtelrose am anfang	shingles early stage	General
gürtelrose endstadium	shingles late stage	General
gürtelrose am kopf behandlung	shingles on head treatment	Localization, Therapy
gürtelrose im auge symptome	shingles in eye treatment	Localization, Symptoms
gürtelrose am bauchnabel	shingles on bellybutton	Localization
gürtelrose handrücken	shingles back of hand	Localization
gürtelrose magenschmerzen	shingles stomach pain	Localization, Symptoms
gürtelrose während der schwangerschaft	shingles during pregnancy	Demographics
ausschlag ähnlich gürtelrose	rash similar shingles	Other Diseases, Symptoms
gürtelrose haut	shingles skin	Symptoms
vzv schwangerschaft	vzv pregnancy	Demographics
herpes zoster differentialdiagnose	herpes zoster differential diagnosis	Other Diseases
gürtelrose seelische ursachen	shingles spiritual causes	Causes
gürtelrose auge ansteckend	shingles eye contagious	Localization, Contagiousness
herpes zoster dauer heilung	herpes zoster duration healing	General
disseminierter zoster	disseminated zoster	Localization
abheilung gürtelrose	healing shingles	General
herpes zoster erreger	herpes zoster pathogen	Causes
rosa zoster	rosa zoster	General
gürtelrose auf der hand	shingles on hand	Localization
bei gürtelrose	with shingles	General
gürtelrose an mehreren stellen gleichzeitig	shingles in more than one place	Localization
gürtelrose im gesicht behandeln	shingles in face treatment	Localization, Therapy
heilpraktiker gürtelrose	naturopath shingles	Therapy
gürtelrose ist die ansteckend	is shingles contagious	Contagiousness

gürtelrose kopf symptome	shingles head symptoms	Localization, Symptoms
herpes zoster im kopf	herpes zoster on head	Localization
generalisierter zoster	generalized zoster	Localization
gürtelrose dauer arbeitsunfähigkeit	shingles duration sick leave	Information
gürtelrose unter dem arm	shingles under arm	Localization
herpes zoster auge symptome	herpes zoster eye symptoms	Localization, Symptoms
herpes zoster nervenschmerzen	herpes zoster nerve pain	Symptoms, Complications
nervenschmerzen herpes	nerve pain herpes	Symptoms, Complications
pfeiffersches drüsenfieber gürtelrose	infectious mononucleosis shingles	Other Diseases
post herpes neuralgie	post herpetic neuralgia treatment	Complications
virustatika herpes zoster	antivirals herpes zoster	Therapy
gürtelrose bluthochdruck	shingles high blood pressure	Other Diseases
gürtelrose haaransatz	shingles hairline	Localization
gürtelrose handinnenfläche	shingles palms	Localization
gürtelrose am bein symptome	shingles on leg symptoms	Localization, Symptoms
gürtelrose wenn man windpocken hatte	shingles when you had chickenpox	Causes
herpes zoster gesäß	herpes zoster buttocks	Localization
hitzepickel oder gürtelrose	heat rash or shingles	Other Diseases
offene gürtelrose	open shingles	General
ursache von gürtelrose	causes for shingles	Causes
krankheitsbild gürtelrose	shingles disease	Symptoms
netdoktor gürtelrose	netdoktor shingles	Information
gürtelrose keine bläschen	shingles no blister	Symptoms
zoster ohr	zoster ear	Localization
gürtelrose an der hüfte	shingles on the hip	Localization
gürtelrose nach grippeimpfung	shingles after flu vaccine	Causes
herpes zoster schmerzmittel	herpes zoster pain medication	Therapy, Symptoms
gürtelrose ansteckend windpocken	shingles contagious chickenpox	Causes, Contagiousness
gürtelrose klein	shingles little	Symptoms
zoster thoracalis	zoster thoracalis	Localization
gürtelrose anfangssymptome	shingles early symptoms	Symptoms
gesichtsrose am auge	facial shingles in eye	Localization
gürtelrose im ohr symptome	shingles in ear symptoms	Localization, Symptoms
kopf herpes	head herpes	Localization

ansteckende gürtelrose	contagious shingles	Contagiousness
gürtelrose und erkältung	shingles and cold	Other Diseases
behandlung gesichtsrose	therapy shingles face	Therapy, Localization
louise hay gürtelrose	louise hay shingles	Information
gürtelrose arm symptome	shingles arm symptoms	Localization, Symptoms
gürtelrose ms	shingles ms	Other Diseases
gürtelrose schulterschmerzen	shingles shoulder pain	Localization, Symptoms
herpes zoster am hals	herpes zoster on neck	Localization
herpes zoster v3	herpes zoster v3	Localization
abgeheilte gürtelrose	healed shingles	General
gürtelrose gliederschmerzen	shingles joint pain	Symptoms
kann gürtelrose mehrmals auftreten	can shingles happen more than once	Information
schmerztherapie bei herpes zoster	pain therapy for herpes zoster	Therapy, Symptoms
gürtelrose dauer ohne behandlung	shingles duration without treatment	Therapy
gürtelrose im hohen alter	shingles in old age	Demographics
gürtelrose in der achselhöhle	shingles in armpit	Localization
gürtelrose starke rückenschmerzen	shingles severe back pain	Localization, Symptoms
gürtelrose und herzbeschwerden	shingles and heart problems	Other Diseases
kann man eine gürtelrose mehrmals bekommen	and you get shingles more than once	Information
leichte form der gürtelrose	mild form of shingles	Symptoms
gürtelrose einseitig	shingles unilateral	Localization
kleinkind gürtelrose	toddler shingles	Symptoms
rezidivierende gürtelrose	recurrent shingles	Information
streifenförmiger ausschlag	striped rash	Symptoms
herpes zoster po polsku	herpes zoster po polsku	General
gürtelrose sexuell übertragbar	shingles sexually transmitted	Contagiousness
gibt es gürtelrose ohne ausschlag	is there shingles without rash	Symptoms
gürtelrose für kinder ansteckend	shingles for children contagious	Contagiousness, Demographics
herpes zoster spätfolgen	herpes zoster late effects	Complications
gürtelrose durch hitze	shingles because of heat	Causes
gürtelrose im alter gefährlich	shingles in old age dangerous	Demographics, Complications
gürtelrose körper	shingles body	Localization

schmerzlinderung bei gürtelrose	pain treatment for shingles	Therapy, Symptoms
ansteckung gürtelrose baby	contagious shingles baby	Contagiousness, Demographics
dauer der gürtelrose	duration of shingles	General
stressbedingte gürtelrose	stress-induced shingles	Causes
gürtelrose am kopf gefährlich	shingles on head dangerous	Localization, Complications
therapie bei herpes zoster	therapy for herpes zoster	Therapy
zoster hand	zoster hand	Localization
herpes zoster am po	herpes zoster on buttocks	Localization
herpes zoster schmerztherapie	herpes zoster pain therapy	Therapy, Symptoms
gürtelrose anfangsstadium an der brust	shingles early stage on chest	Localization
wenn man windpocken hatte kann man dann gürtelrose bekommen	when one had chickenpox can one get shingles later	Causes
gürtelrose mit 25	shingles at 25	Demographics
gürtelrose ohne symptome	shingles without symptoms	Symptoms
windpocken gürtelrose ansteckung	chickenpox shingles contagiousness	Contagiousness
gürtelrose heilungsverlauf	shingles healing progression	General
zostereffloreszenzen	zoster skin conditions	Symptoms
gürtelrose ansteckung über dritte	shingles transmission through third party	Contagiousness
ansteckungszeit bei gürtelrose	incubation period for shingles	Contagiousness
gürtelrose am auge symptome	shingles in eye symptoms	Localization, Symptoms
gürtelrose griechisch	shingles greek	General
herpes zoster neuralgie therapie	herpes zoster neuralgia therapy	Symptoms, Complications, Therapy
schmerztherapie herpes zoster	pain therapy herpes zoster	Therapy, Symptoms
gürtelrose unterm auge	shingles under eye	Localization
kann man gürtelrose bekommen wenn man keine windpocken hatte	can one get shingles if one has not had chickenpox	Causes
windpocken gürtelrose herpes	chickenpox shingles herpes	Causes
zoster sine herpete therapie	zoster sine herpete therapy	Symptoms, Therapy
gesichtsrose ansteckungsgefahr	facial shingles contagion risk	Localization, Contagiousness
gürtelrose woher kommt das	shingles where does it come from	Causes
gürtelrose hals symptome	shingles neck symptoms	Localization, Symptoms

gürtelrose im brustbereich	shingles on chest	Localization
ist zoster ansteckend	is shingles contagious	Contagiousness
gürtelrose als kind	shingles as child	Demographics
gürtelrose ursachen und risikofaktoren	shingles causes and risk factors	Causes
sonne bei gürtelrose	sun during shingles	Causes
gürtelrose hygienemaßnahmen	shingles hygiene measures	Contagiousness
herpes zoster fieber	herpes zoster fever	Symptoms
mit gürtelrose zur arbeit	going to work with shingles	Contagiousness
herpes zoster ansteckung baby	herpes zoster contagiousness baby	Contagiousness, Demographics
ist gürtelrose	is shingles	General
gürtelrose dauer der krankheit	shingles duration of disease	General
symptome gürtelrose kopf	symptoms shingles head	Symptoms, Localization
gesichtsgürtelrose symptome	facial shingles symptoms	Symptoms, Localization
gürtelrose und psyche	shingles and psyche	Causes
gürtelrose brustkrebs	shingles breast cancer	Other Diseases
gürtelrose isolation	shingles isolation	Information
gürtelrose rippenschmerzen	shingles rib pain	Localization, Symptoms
gürtelrose symptome rückenschmerzen	shingles symptoms back pain	Symptoms, Localization
neuralgie nach gürtelrose	neuralgia after shingles	Complications
rezidivierender herpes zoster	recurrent herpes zoster	Information
zoster v2	zoster v2	Localization
blutwerte gürtelrose	blood values shingles	Information
geschlossene gürtelrose	closed shingles	General
gürtelrose hausarzt	shingles general practitioner	Therapy
gürtelrose kinder symptome	shingles children symptoms	Demographics
herpes zoster schulter	herpes zoster shoulder	Localization
natürliche hausmittel gegen gürtelrose	natural home remedies for shingles	Therapy
gürtelrose ausschlag ohne bläschen	shingles rash without blisters	Symptoms
gürtelrose lebensbedrohlich	shingles life threatening	Complications
gürtelrose stationär behandeln	shingles inpatient treatment	Therapy
ursache herpes zoster	causes herpes zoster	Causes
gürtelrose ähnlicher ausschlag	shingles similar rash	Symptoms
kann gürtelrose an mehreren stellen gleichzeitig auftreten	can shingles occur on more than one localization at once	Localization
kopfrose kopfhaut	shingles head scalp	Localization
gürtelrose immer schmerzhaft	shingles always painful	Symptoms

gürtelrose mehrere stellen	shingles many localizations	Localization
herpes zoster laborwerte	herpes zoster lab values	Information
gürtelrose achselhöhle	shingles armpit	Localization
hausmittel gürtelrose behandlung	home remedy shingle therapy	Therapy
zoster ohne ausschlag	zoster without rash	Symptoms
gürtelrose achselbereich behandlung	shingles armpit treatment	Localization, Therapy
herpes zoster sine	herpes zoster sine	Symptoms
gürtelrose nach geburt	shingles after childbirth	Causes
gürtelrose tumor	shingles tumor	Other Diseases
kann man gürtelrose am bein bekommen	can you get shingles on leg	Localization
zinksalbe gegen gürtelrose	zinc ointment for shingles	Therapy
gürtelrose bei kindern trotz windpockenimpfung	shingles in children despite chickenpox vaccine	Demographics, Vaccine
gürtelrose im rachen	shingles in pharynx	Localization
lippenherpes gürtelrose	lip herpes shingles	Other Diseases
gürtelrose ansteckung neugeborene	shingles contagiounesness newborn	Contagiousness, Demographics
gürtelrose im auge ansteckend	shingles in eye contagious	Localization, Contagiousness
gürtelrose bei männern	shingles in men	Demographics
gürtelrose juckt beim abheilen	shingles itches when healing	Symptoms
gürtelrose nervenentzündung	shingles nerve inflammation	General
gürtelrose oder nesselsucht	shingles or urticaria	Information
herpes zoster im blut nachweisbar	shingles detectable in blood	Information
gürtelrose schmerzhaft	shingles painful	Symptoms
feuchtblattern gürtelrose	chickenpox shingles	Causes
gürtelrose am körper	shingles on body	Localization
gürtelrose an beinen	shingles on legs	Localization
herpes zoster nacken	herpes zoster neck	Localization
kann gürtelrose windpocken auslösen	can shingles cause chickenpox	Causes
gürtelrose oberschenkel innenseite	shingles inner thigh	Localization
nesselsucht oder gürtelrose	shingles or urticaria	Other Diseases
vorbeugung gürtelrose	prevent shingles	Therapy
gürtelrose geschlechtskrankheit	shingles sexually transmitted disease	Other Diseases
kopfgürtelrose ansteckend	head shingles contagious	Contagiousness
zoster ohne bläschen	zoster without blisters	Symptoms
gesichtsrose erste symptome	facial shingles first symptoms	Localization, Symptoms

gürtelrose mehrmals im leben	shingles many times during lifetime	Information
gürtelrose nierenschmerzen	shingles kidney pain	Symptoms
gürtelrose rachen	shingles pharynx	Localization
herpes ausschlag im gesicht	herpes rash in face	Localization, Symptoms
olivenblattextrakt gürtelrose	olive leaf extract shingles	Therapy
ursachen einer gürtelrose	causes for shingles	Causes
virostatika herpes zoster	antivirals herpes zoster	Therapy
gürtelrose anfangsstadium am rücken	shingles early stage on back	Localization
gürtelrose dahlke	shingles dahlke	Information
gürtelrose schambereich	shingles genital area	Localization
ist gürtelrose schmerzhaft	is shingles painful	Symptoms
gürtelrose tumormarker	shingles tumor marker	Other Diseases
herpes zoster sta je to	herpes zoster sta je to	General
mit gürtelrose in die schule	going to school with shingles	Contagiousness
schmerztherapie nach gürtelrose	pain therapy after shingles	Therapy, Symptoms, Complications
tk gürtelrose	tk shingles	Information
behandlung gürtelrose im gesicht	therapy shingles face	Therapy, Localization
gürtelrose am hals ohne schmerzen	shingles on neck without pain	Localization, Symptoms
gürtelrose dauer erkrankung	shingles duration diseases	General
gürtelrose durch zeckenbiss	shingles through tick bite	Causes
herpes zoster im genitalbereich	shingles in genital area	Localization
herpes zoster po	shingles buttocks	Localization
hirnhautentzündung durch gürtelrose	meningitis because of shingles	Complications
gürtelrose arbeitsunfähigkeit	shingles sick leave	Information
gürtelrose ausbruch	shingles break out	General
gürtelrose autoimmunerkrankung	shingles autoimmunedisease	Other Diseases
gürtelrose begleiterscheinungen	shingles symptoms	Symptoms
gürtelrose geschlechtsverkehr	shingles sexual intercouse	Information
gürtelrose symptome bauch	shingles symptoms abdomen	Localization, Symptoms
herpes am ohr gefährlich	herpes ear dangerous	Localization, Complications
herpes zoster am arm	herpes zoster on arm	Localization
gürtelrose darm	shingles intestine	Localization
gürtelrose ohne bläschenbildung	shingles without blisters	Symptoms
auslöser von gürtelrose	trigger for shingles	Causes

gürtelrose am steißbein	shingles on coccyx	Localization
herpes zoster auslöser	herpes zoster trigger	Causes
zoster bläschen	zoster blisters	Symptoms
zoster sine herpete ansteckend	zoster sine herpete contagious	Symptoms, Contagiousness
gürtelrose im bein	shingles on leg	Localization
heilungsprozess gürtelrose	healing process shingles	General
herpes zoster am ohr	herpes zoster on ear	Localization
übertragung von gürtelrose	transmission of shingles	Contagiousness
gürtelrose gesichtsrose	shingles face	Localization
gürtelrose ist ansteckend	is shingles contagious	Contagiousness
gürtelrose nebenerscheinungen	shingles symptoms	Symptoms
herpes zoster fußsohle	herpes zoster sole feet	Localization
gürtelrose schwangerschaft ansteckung	shingles pregnancy contagiousness	Demographics, Contagiousness
gürtelrose und baby	shingles and baby	Demographics
hirnhautentzündung gürtelrose	meningitis shingles	Complications
körperpflege bei gürtelrose	body care during shingles	Therapy
ursachen herpes zoster	causes herpes zoster	Causes
gürtelrose am hals symptome	shingles neck symptoms	Localization
gürtelrose an verschiedenen stellen	shingles on different body parts	Localization
herpes zoster augenlid	herpes zoster eyelid	Localization
gesichtsrose kinder	facial shingles children	Localization, Demographics
herpes zoster kinder therapie	facial shingles children therapy	Demographics, Therapy
infektion gürtelrose	infection shingles	General
ist gürtelrose ansteckend für schwangere	is shingles contagious for pregnant women	Contagiousness, Demographics
kann man sich mit gürtelrose anstecken wenn man windpocken hatte	can one infect themselves with shingles when they have had chickenpox	Contagiousness, Causes
taubheitsgefühl bei gürtelrose	numbness during shingles	Symptoms
gürtelrose heilmittel	shingles treatments	Therapy
gürtelrose robert franz	shingles robert franz	Information
gürtelrose zovirax	shingles zovirax	Therapy
gürtelrose schmerzen im bein	shingles pain in leg	Symptoms, Localization
gürtelrose windpocken ansteckung	shingles chickenpox contagion	Causes, Contagiousness
gürtelrose an den augen	shingles in the eyes	Localization
gürtelrose hintern	shingles rear	Localization

zoster schmerztherapie	zoster pain therapy	Therapy, Symptoms
gürtelrose im jungen alter	herpes zoster in young age	Demographics
herpes zoster heilung	herpes zoster healing	General
behandlung von herpes zoster	treatment of herpes zoster	Therapy
gürtelrose auf der zunge	shingles on tongue	Localization
gürtelrose krankheitsbild	shingles disease	General
zoster in der schwangerschaft	zoster in pregnany	Demographics
an welchen stellen kann gürtelrose auftreten	on which body parts can shingles develop	Localization
herpes zoster c2	herpes zoster c2	Localization
bei gürtelrose ins krankenhaus	go to hospital with shingles	Therapy
gürtelrose augenbraue	shingles eyebrow	Localization
gürtelrose innenliegend	internal shingles	Localization
ständig gürtelrose	always shingles	Information
gürtelrose körperstellen	shingles body parts	Localization
gürtelrose ursache ansteckung	shingles cause contagiousness	Causes, Contagiousness
gürtelrose windpockenimpfung	shingles chickenpox vaccine	Causes, Vaccine
herpes an der schulter	herpes on shoulder	Localization
gürtelrose in der stillzeit	shingles during breastfeeding	Demographics
herpes sine	herpes sine	Symptoms
brivudin herpes zoster	brivudin herpes zoster	Therapy
gürtelrose rheuma	shingles rheuma	Other Diseases
naturheilmittel gegen gürtelrose	natural remedy for shingles	Therapy
schwere gürtelrose	severe shingles	Symptoms
gesichtsrose schwangerschaft	facial shingles pregnancy	Localization, Demographics
gürtelrose erkrankung	shingles disease	General
gürtelrose von alleine weg	shingles goes away on its own	Therapy
symptome gürtelrose am kopf	symptoms shingles on head	Symptoms, Localization
verlauf einer gürtelrose	disease course of shingles	General
bekommt man gürtelrose wenn man windpocken hatte	does one get shingles when one had chickenpox	Causes
dauer gürtelrose gesicht	duration shingles face	Localization
gürtelrose kann man sterben	shingles can one die	Complications
symptome gürtelrose hals	symptoms shingles neck	Symptoms, Localization
gürtelrose facharzt	shingles specialist	Therapy
gürtelrose an hand	shingles on hand	Localization
gürtelrose ansteckungsgefahr schwangerschaft	shingles risk of contagion pregnancy	Contagiousness, Demographics

gürtelrose bei krebs	shingles cancer	Other Diseases
gürtelrose schmerzen arm	shingles pain arm	Symptoms, Localization
gürtelrose stressbedingt	shingles stress-induced	Causes
gürtelrose und schwanger	shingles and pregnant	Demographics
zoster am kopf	zoster on head	Localization
gürtelrose durch psychischen stress	shingles because of psychological stress	Causes
gürtelrose gesäß ansteckend	shingles buttocks contagious	Localization, Contagiousness
gürtelrose zeh	shingles toe	Localization
ursache gürtelrose rücken	causes shingles back	Causes, Localization
gürtelrose selbstheilung	shingles self treatment	Therapy
gürtelrose vererbbar	shingles inherited	Causes
masern und gürtelrose	measles and shingles	Other Diseases
schmerzbehandlung bei gürtelrose	pain treatment for shingles	Therapy, Symptoms
ausschlag ähnlich wie gürtelrose	rash similar to shingles	Other Diseases, Symptoms
facharzt für gürtelrose	specialist for shingles	Therapy
gürtelrose am rücken schmerzen	shingles on back pain	Localization, Contagiousness, Pain
gürtelrose säugling ansteckung	shingles infant contagiousness	Demographics, Contagiousness
herpes zoster und herpes simplex	herpes zoster and herpes simplex	Other Diseases
naturheilmittel bei gürtelrose	natural remedy for shingles	Therapy
gürtelrose bläschen behandeln	shingles blister treatment	Therapy, Symptoms
gürtelrose im gesicht ohne ausschlag	shingles in face without rash	Localization, Symptoms
gürtelrose nervenbahnen	shingles nerve pathways	General
welche symptome bei gürtelrose	which symptoms for shingles	Symptoms
gürtelrose am bein anfangsstadium	shingles on leg early stages	Localization
gürtelrose erreger windpocken	shingles pathogen chickenpox	Causes
heilungsverlauf gürtelrose	healing process shingles	General
gürtelrose auf persisch	shingles in persian	General
gürtelrose kinderwunsch	shingles fertility wish	Demographics
gürtelrose kopfhaut symptome	shingles scalp symptoms	Localization, Symptoms
gürtelrose mehrmals bekommen	shingles more than once	Information
stiko empfehlung gürtelrose	stiko recommendation shingles	Information, Vaccine

gesichtsrose augen	face shingles eyes	Localization
gürtelrose im ohr ansteckend	shingles in ear contagious	Localization, Contagiousness
gürtelrose unerträgliche schmerzen	shingles unbearable pain	Symptoms
herpes rose	herpes rose	General
virostatikum herpes zoster	antivirals herpes zoster	Therapy
chronischer herpes zoster	chronic herpes zoster	Complications
erneute gürtelrose	recurrent shingles	Information
gürtelrose kleine stelle	shingles small area	Symptoms
herpes zoster gesicht therapie	herpes zoster face therapy	Localization, Therapy
kann man sich mit gürtelrose anstecken	can one infect themselves with shingles	Contagiousness
krebs gürtelrose	cancer shingles	Other Diseases
zoster im ohr	zoster in ear	Localization
gürtelrose am rücken ansteckend	shingles on back contagious	Localization, Contagiousness
gürtelrose hautarzt oder hausarzt	shingles dermatologist or general practitioner	Therapy
herpes und gürtelrose gleiche viren	herpes and shingles same virus	General
herpes zoster sonne	herpes zoster sun	Causes
zoster im mund	zoster in mouth	Localization
ansteckungsgefahr herpes zoster	risk of contagion herpes zoster	Contagiousness
gürtelrose am oberkörper	shingles on upper body	Localization
herpes zoster infektiös	herpes zoster infectious	Contagiousness
nesselsucht gürtelrose	hives shingles	Other Diseases
gürtelrose säugling gefährlich	shingles infant dangerous	Demographics, Complications
gürtelrose gesicht kind	shingles face child	Localization, Demographics
gürtelrose meerwasser	shingles sea water	Therapy
gürtelrose narben behandeln	shingles scars treatment	Complications, Therapy
schwangerschaft und gürtelrose	pregnancy and shingles	Demographics
gürtelrose handfläche	shingles palms	Localization
gürtelrose nur an einer stelle	shingles only on one place	Symptoms
herpes zoster ohne hautausschlag	herpes zoster without rash	Symptoms
schmerzen nach herpes zoster	pain after herpes zoster	Complications
schmerztherapie zoster	pain therapy zoster	Therapy, Symptoms
verlauf gürtelrose rücken	progression shingles back	Localization

gürtelrose ansteckung säugling	shingles contagiousnes infant	Contagiousness, Demographics
gürtelrose bei ms	shingles with ms	Other Diseases
gürtelrose im gesicht therapie	shingles in face therapy	Localization, Therapy
gürtelrose virostatika	shingles antivirals	Therapy
gürtelrose wie gefährlich	shingles how dangerous	Complications
ms gürtelrose	ms shingles	Other Diseases
stiko empfehlung herpes zoster	stiko recommendation herpes zoster	Information, Vaccine
trockene gürtelrose	dry shingles	Symptoms
disseminierter herpes zoster	disseminated herpes zoster	Localization
gürtelrose durch alkohol	shingles because of alcohol	Causes
gürtelrose für wen ansteckend	shingles for whom contagious	Contagiousness
gürtelrose nach innen	shingles internal	Localization
zoster im auge	zoster in eye	Localization
gürtelrose am arm ohne ausschlag	shingles on arm without rash	Localization, Symptoms
gürtelrose leberwerte	shingles liver values	Information
gürtelrose symptome kinder	shingles children symptoms	Symptoms, Demographics
gürtelrose ältere menschen	shingles elderly	Demographics
kann man die gürtelrose mehrmals bekommen	can one get shingles more than once	Information
kann man gürtelrose bekommen wenn man schon windpocken hatte	can one get shingles they had already had chickenpox	Causes
lokaltherapie herpes zoster	local therapy herpes zoster	Therapy
windpocken nach gürtelrose	chickenpox after shingles	Causes
zoster sine zoster	zoster sine zoster	Symptoms
gesichtsrose ohne schmerzen	shingles without pain	Localization, Symptoms
herpes zoster zoster	herpes zoster zoster	General
kann man herpes zoster mehrmals bekommen	can one get herpes zoster more than once	Information
ansteckung gürtelrose schwangerschaft	contagiousness shingles pregnancy	Contagiousness, Demographics
gürtelrose genitalien	shingles genitals	Localization
gürtelrose lebensgefahr	shingles life threatening	Complications
gürtelrose schmerzen in der brust	shingles chest pain	Symptoms, Localization
zoster kopf	zoster head	Localization
gürtelrose bauch symptome	shingles belly symptoms	Localization, Symptoms
gürtelrose im gesicht auge	shingles in face eye	Localization

gürtelrose kopf dauer	shingles head duration	Localization
gürtelrose virustatika	shingles antivirals	Therapy
hautkrankheit rosengürtel	skin disease shingles	Symptoms
herpes zoster auge therapie	herpes zoster eye therapy	Localization, Therapy
starke schmerzen bei gürtelrose	severe pain during shingles	Symptoms
zoster neuralgie symptome	zoster neuralgia symptoms	Complications, Symptoms
anfangssymptome gürtelrose	early symptoms shingles	Symptoms
gürtelrose analbereich	shingles anal region	Localization
herpes zoster hygienemaßnahmen	herpes zoster hygiene measures	Contagiousness
behandlung post zoster neuralgie	therapy post zoster neuralgia	Therapy, Complications
gürtelrose am gesicht	shingles in face eye	Localization
gürtelrose am kopf schmerzen	shingles on head pain	Localization, Symptoms
gürtelrose ausschlag geht nicht weg	shingles rash does not go away	Symptoms
gürtelrose unsichtbar	shingles invisible	Symptoms
gürtelrose unter achsel	shingles armpit	Localization
gürtelrose während schwangerschaft	shingles during pregnancy	Demographics
gürtelrose am fussrücken	shingles on instep	Localization
gürtelrose am grossen zeh	shingles on big toe	Localization
gürtelrose ansteckend für geimpfte kinder	shingles contagious for vaccinated children	Contagiousness, Vaccine, Demographics
herpes zoster am gesäß	herpes zoster on buttocks	Localization
herpes zoster ansteckung schwangerschaft	herpes zoster contagious pregnancy	Contagiousness, Demographics
herpes zoster mehrfach	herpes zoster many times	Information
kann ich gürtelrose bekommen wenn ich windpocken hatte	can i get shingles when i had chickenpox	Causes
rheuma und gürtelrose	rheuma and shingles	Other Diseases
gürtelrose am hals gefährlich	shingles on neck dangerous	Localization, Complications
gürtelrose im körper	shingles in body	Localization
gürtelrose schmerzen wie muskelkater	shingles pain like sore muscles	Symptoms
johanniskrautöl bei gürtelrose	st. john's wort for shingles	Therapy
nach gürtelrose schlapp	after shingles exhausted	Complications
anfang von gürtelrose	beginning of shingles	General
gürtelrose auge behandlung	shingles eye treatment	Localization, Therapy
schmerzen nach der gürtelrose	pain after shingles	Complications

symptome gürtelrose im gesicht	symptoms shingles in face	Symptoms, Localization
beschwerden bei gürtelrose	symptoms shingles	Symptoms
gürtelrose ansteckungsgefahr baby	shingles risk of contagion baby	Contagiousness, Demographics
gürtelrose in den augen	shingles in the eyes	Localization
kann man gürtelrose im blut nachweisen	can one detect shingles in blood	Information
taubheitsgefühl nach gürtelrose	numbness after shingles	Symptoms
gesichtsrose bei kindern	facial shingles in children	Localization, Demographics
gürtelrose an brust	shingles on chest	Localization
gürtelrose gesicht auge	shingles face eye	Localization
gürtelrose gesundheitsamt	shingles department of health	Information
gürtelrose im mundbereich	shingles mouth area	Localization
gürtelrose und rückenschmerzen	shingles and back pain	Symptoms, Localization
naturheilmittel gürtelrose	natural remedies shingles	Therapy
gürtelrose am kopf ohne ausschlag	shingles on head without rash	Localization, Symptoms
schmerzen herpes zoster	pain herpes zoster	Symptoms
behandlung zoster	therapy zoster	Therapy
gürtelrose durch seelischen stress	shingles durch spiritual stress	Causes
gürtelrose ss	shingles pregnancy week	Demographics
herpes zoster am rücken	herpes zoster back	Localization
differentialdiagnose gürtelrose	differential diagnosis shingles	Other Diseases
gürtelrose im hals innen	shingles in neck internal	Localization
gürtelrose im schambereich	shingles in genital area	Localization
gürtelrose ischiasnerv	shingles sciatic nerve	Localization
gürtelrose nach brustkrebs	shingles after breast cancer	Causes
herpes zoster auge ansteckung	herpes zoster eye contagiousness	Localization, Contagiousness
rückenschmerzen durch gürtelrose	back pain because of shingles	Symptoms, Localization
fachklinik gürtelrose	specialist clinic shingles	Therapy
gürtelrose schwangerschaft behandlung	shingles pregnancy treatment	Demographics, Therapy
haarausfall nach gürtelrose	hairloss after shingles	Complications
herpes zoster ophthalmicus therapie	herpes zoster ophthalmicus therapy	Localization, Therapy
herpes zoster paracetamol	herpes zoster paracetamol	Therapy
gürtelrose ohr symptome	shingles ear symptoms	Localization, Symptoms

kann man gürtelrose häufiger bekommen	can one get shingles more than once	Information
meldepflicht herpes zoster	reporting requirements herpes zoster	Information
virostatika bei gürtelrose	antivirals for shingles	Therapy
hausmittel gegen herpes zoster	home remedy for herpes zoster	Therapy
kann man an einer gürtelrose sterben	can one die because of shingles	Complications
gürtelrose am hals ansteckend	shingles on neck contagious	Localization, Contagiousness
gürtelrose bei stress	shingles with stress	Causes
gürtelrose geht von alleine weg	shingles goes away on its own	Complications
gürtelrose immer ansteckend	shingles always contagious	Contagiousness
gürtelrose pfeiffersches drüsenfieber	shingles infectious mononucleosis	Other Diseases
postzosterische neuralgie symptome	posterzoster neuralgia symptoms	Complications, Symptoms
zoster ausschlag	zoster rash	Symptoms
folgen der gürtelrose	complications of shingles	Complications
welche schmerzen bei gürtelrose	which pain for shingles	Symptoms
deutsche schmerzliga gürtelrose	deutsche schmerzliga shingles	Information, Complications
gürtelrose im mund hausmittel	shingles in mouth home remedy	Localization, Therapy
gürtelrose pflanzlich behandeln	shingles herbal treatment	Therapy
gürtelrose und fieber	shingles and fever	Symptoms
halsschmerzen bei gürtelrose	throat pain with shingles	Symptoms, Localization
herpes am oberarm	herpes on upper arm	Localization
herpes gürtelrose gesicht	herpes shingles face	Localization
robert koch institut gürtelrose	robert koch institut shingles	Information
gürtelrose wo am körper	shingles where on body	Localization
herpes zoster darm	herpes zoster intestines	Localization
nach gürtelrose taubheitsgefühl	after shingles numbness	Complications
rezidivierende gürtelrose ursache	recurrent shingles cause	Information, Causes
gürtelrose am kopfhaut	shingles on scalp	Localization
gürtelrose herpes simplex	shingles herpes simplex	Other Diseases
gürtelrose jugendliche	shingles young people	Demographics
gürtelrose oder mückenstiche	shingles or mosquito bite	Other Diseases
gürtelrose schafblattern	shingles chickenpox	Causes
gürtelrose schmerzen rücken	shingles pain back	Symptoms, Localization
gürtelrose und stress	shingles and stress	Causes
hautpflege gürtelrose	skin care shingles	Therapy

herpes am ohr kind	herpes on ear child	Localization, Demographics
herpes zoster folgen	herpes zoster consequences	Complications
herpes zoster gesicht behandlung	herpes zoster face therapy	Localization, Therapy
ohrenschmerzen bei gürtelrose	ear pain with shingles	Localization, Symptoms
gürtelrose im kopf symptome	shingles on head symptoms	Localization, Symptoms
gürtelrose schulter nacken	shingles shoulder nape	Localization
herzprobleme durch gürtelrose	heart problems because of shingles	Complications
ist gürtelrose immer ansteckend	is shingles always contagious	Contagiousness
kann gürtelrose mehrfach auftreten	can you get shingles more than once	Information
nach gürtelrose immer noch schmerzen	after shingles still pain	Complications
nervenschmerzen nach herpes zoster	nerve pain after herpes zoster	Complications
zoster bei kindern	zoster in children	Demographics
gürtelrose ansteckend für erwachsene	shingles contagious for adults	Contagiousness, Demographics
johanniskrautöl gürtelrose	st. john's wort shingles	Therapy
verlauf herpes zoster	disease progression herpes zoster	General
dauer herpes zoster	duration herpes zoster	General
gürtelrose höhepunkt	shingles peak	General
gürtelrose juckt nicht	shingles does not itch	Symptoms
hautausschlag ähnlich gürtelrose	rash similar to shingles	Other Diseases, Symptoms
herpes auf rücken	herpes on back	Localization
hygienemaßnahmen bei gürtelrose	hygiene measures for shingle	Contagiousness
windpocken zoster	chickenpox zoster	Causes
gürtelrose psychische ursachen	shingles psychological causes	Causes
gürtelrose schmerzen nach abheilung	shingles pain after healing	Symptoms, Complications
gürtelrose stechende schmerzen	shingles stabbing pain	Symptoms
erreger der gürtelrose	pathogen for shingles	Causes
formen der gürtelrose	types of shingles	General
gürtelrose a	shingles a	General
gürtelrose einzelne bläschen	shingles individual blisters	Symptoms
gürtelrose kopf verlauf	shingles head disease progression	Localization
gürtelrose schmerzen am ganzen körper	shingles pain on entire body	Symptoms, Localization
herpes sine herpete symptoms	herpes sine herpete symptoms	Symptoms
herpes zoster verlauf dauer	herpes zoster progression duration	General

schmerzen bei gürtelrose behandeln	pain shingles treatment	Therapy, Symptoms
gürtelrose gefährlich für kleinkinder	shingles dangerous toddlers	Complications, Demographics
gürtelrose schmerzlinderung	shingles pain treatment	Symptoms, Therapy
herpes zoster natürlich behandeln	herpes zoster natural treatment	Therapy
kopfrose therapie	shingles head treatment	Localization, Therapy
therapie zoster neuralgie	therapy zoster neuralgia	Therapy, Complications, Symptoms
ursache gürtelrose im gesicht	causes shingles in face	Causes, Localization
bauchschmerzen bei gürtelrose	abdomen pain in shingles	Symptoms
gürtelrose bandscheibenvorfall	shingles herniated disc	Other Diseases
gürtelrose kann ich mein kind anstecken	shingles can i infect my child	Contagiousness, Demographics
gürtelrose kita	shingles daycare	Contagiousness
gürtelrose schwache form	shingles mild form	Symptoms
habe ich gürtelrose	do i have shingles	General
herpes zoster gesichtslähmung	herpes zoster facial paralysis	Localization, Symptoms
schmerzmittel bei nervenschmerzen gürtelrose	pain medication for nerve pain shingles	Therapy, Symptoms, Complications
verlauf gürtelrose bauch	progression shingles belly	Localization
gürtelrose auf der nase	shingles on the nose	Localization
gürtelrose frühsymptome	shingles early symptoms	Symptoms
gürtelrose nesselsucht	shingles urticaria	Other Diseases
herpes nervenentzündung	herpes nerve inflammation	General
steckt gürtelrose an	is shingles contagious	Contagiousness
zoster bein	zoster leg	Localization
ausbruch gürtelrose	outbreak shingles	General
gürtelrose um den mund	shingles around the mouth	Localization
gürtelrose übertragungswege	shingles modes of transmission	Contagiousness
ist gürtelrose ansteckend für babys	is shingles contagious for babies	Contagiousness, Demographics
kommt gürtelrose immer wieder	does shingles always come back	Information
leichte gürtelrose symptome	mild shingles symptoms	Symptoms
lidocain pflaster zoster	lidocaine plaster zoster	Therapy
lippenherpes und gürtelrose gleichzeitig	lip herpes and shingles simultaneously	Other Diseases
schon wieder gürtelrose	again shingles	Information

chronische schmerzen nach gürtelrose	chronic pain after shingles	Complications
gürtelrose an schulter	shingles on shoulder	Localization
gürtelrose meldepflichtig 2018	shingles reportable 2018	Information
gürtelrose schlapp	shingles exhausted	Symptoms
herpes zoster s	herpes zoster s	Localization
kann man mehrmals an gürtelrose erkranken	can one get shingles more than once	Information
mehrfach gürtelrose	many times shingles	Information
gürtelrose fussrücken	shingles instep	Localization
gürtelrose kinder ansteckend	shingles children contagious	Demographics, Contagiousness
gürtelrose laborwerte	shingle lab values	Information
gürtelrose übertragung durch dritte	shingles transmission through third party	Contagiousness
herpes generalisatus	herpes generalisatus	Localization
behandlung einer gürtelrose	therapy shingles	Therapy
gürtelrose anfangsstadium rücken	shingles early stage back	Localization
gürtelrose blutdruck	shingles blood pressure	Other Diseases
hautkrankheit gürtelrose	skin disease shingles	Symptoms
immer wiederkehrende gürtelrose	recurrent shingles	Information
ist gürtelrose eine nervenkrankheit	is shingles a nerve disease	General
sine herpete zoster	sine herpete zoster	Symptoms
gürtelrose am bauch symptome	shingles on belly symptoms	Localization, Symptoms
gürtelrose erneute erkrankung	shingles new disease	Information
gürtelrose unter dem fuß	shingles under foot	Localization
narben von gürtelrose	scars because of shingles	Complications
ansteckung mit gürtelrose	transmission of shingles	Contagiousness
herpes zoster beidseitig	herpes zoster bilateral	Localization
gürtelrose am hals wie lange krankgeschrieben	shingles on leg how long sick leave	Localization, Information
gürtelrose bei geimpften kindern	shingles in vaccinated children	Demographics, Vaccine
herpes zoster unbehandelt	herpes zoster untreated	Therapy
kokosöl bei gürtelrose	coconut oil for shingles	Therapy
symptome zoster	symptoms zoster	Symptoms
tens herpes zoster	tens herpes zoster	Therapy
zoster am bein	zoster on leg	Localization
gürtelrose rippenbogen	shingles rib cage	Localization
gürtelrose verlauf der krankheit	shingles disease progression	General

herpes zoster ohrenschmerzen	herpes zoster ear pain	Localization, Symptoms
symptome gürtelrose ohne ausschlag	symptoms shingles without rash	Symptoms
gürtelrose sonnenbrand	shingles sunburn	Causes
herpes am kopf ansteckend	herpes on head contagious	Localization, Contagiousness
post zoster neuralgie arbeitsunfähig	post zoster neuralgia sick leave	Complications, Information
poster zoster neuralgie	poster zoster neuralgia	Complications
zoster verlauf	zoster progression	General
awmf zoster	awmf zoster	Information
behandlungsdauer gürtelrose	therapy duration shingles	Therapy
gibt es gürtelrose im gesicht	is there shingles in the face	Localization
gürtelrose schnell heilen	shingles fast healing	Therapy
ist gürtelrose ansteckend ja oder nein	is shingles contagious yes or no	Contagiousness
symptome gürtelrose nacken	symptoms shingles neck	Symptoms, Localization
verlauf der gürtelrose	disease progression shingles	General
anfangsstadium gürtelrose kind	early stages shingles child	Demographics
ansteckung gürtelrose kleinkind	risk of contagion shingles toddler	Contagiousness, Demographics
gürtelrose immer wieder neue bläschen	shingles always new blister	Information, Symptoms
gürtelrose infektiös	shingles infectious	Contagiousness
gürtelrose oder allergie	shingles or allery	Other Diseases
postherpetische neuralgie symptome	postherpetic neuralgia symptoms	Symptoms, Complications
virustatika bei gürtelrose	antiviral for shingles	Therapy
dauer von gürtelrose	duration of shingles	General
gürtelrose bei frauen	shingles in women	Demographics
gürtelrose bei säuglingen	shingles in infants	Demographics
gürtelrose nach chemo	shingles after chemo	Causes
gürtelrose trockene haut	shingles dry skin	Symptoms
behandlung gürtelrose rücken	therapy shingles back	Therapy, Localization
gürtelrose am po und bein	shingles on buttocks and leg	Localization
gürtelrose ansteckungszeitraum	shingles incubation period	Contagiousness
gürtelrose im genitalbereich symptome	shingles in genital area symptoms	Localization, Symptoms
gürtelrose mundwinkel	shingles corner of mouth	Localization
hautausschlag ähnlich wie gürtelrose	rash like shingles	Other Diseases, Symptoms
herpes zoster c3	herpes zoster c3	Localization

kann man gürtelrose mehrmals haben	can you get shingles more than once	Information
leichte form von gürtelrose	mild form of shingles	Symptoms
gürtelrose an händen	shingles on hands	Localization
zoster hals	zoster neck	Localization
gürtelrose nach schwangerschaft	shingles after pregnancy	Demographics
gürtelrose ohne ausschlag ansteckend	shingles without rash contagious	Symptoms, Contagiousness
gürtelrose wegen stress	shingles because of stress	Causes
herpes zoster narben	herpes zoster scars	Complications
herpes zoster nasenspitze	herpes zoster tip of nose	Localization
krankheitsverlauf gürtelrose dauer	disease progression shingles duration	General
symptome gürtelrose auge	symptoms shingles eye	Symptoms, Localization
allgemeinbefinden bei gürtelrose	general well-being shingles	Symptoms
gürtelrose baby gefährlich	shingles baby dangerous	Demographics, Complications
herpes auf dem arm	herpes on the arm	Localization
nervenschmerzen durch herpes	nerve pain because of herpes	Symptoms, Complications
post zoster neuralgie diagnose	post zoster neuralgia diagnosis	Complications
schmerzen bei herpes zoster	pain in herpes zoster	Symptoms
symptome gürtelrose bei erwachsenen	symptoms shingles adults	Symptoms, Demographics
symptome kopfrose	symptoms shingles head	Symptoms, Localization
ähnlich gürtelrose	similar shingles	Other Diseases
cortisonsalbe bei gürtelrose	cortison cream in shingles	Therapy
gürtelrose akupunkturpunkte	shingles acupuncture points	Therapy
gürtelrose dauer und verlauf	shingles duration and progression	General
gürtelrose im schritt	shingles in groin	Localization
gürtelrose kind gesicht	shingles child face	Demographics, Localization
gürtelrose schläfe	shingles temples	Localization
gürtelrose und neugeborene	shingles and newborns	Demographics
gürtelrose ähnliche symptome	shingles similar symptoms	Symptoms
herpes zoster in schwangerschaft	herpes zoster in pregnancy	Demographics
herpes zoster wiederholter ausbruch	herpes zoster recurrent outbreak	Information
nervenschmerzen durch gürtelrose	nerve pain because of shingles	Symptoms, Complications
verdeckte gürtelrose	hidden shingles	Symptoms

ansteckung von gürtelrose	transmission of shingles	Contagiousness
eine gürtelrose	a shingles	General
gürtelrose am bein anfang	shingles on leg beginning	Localization
virostatika gegen gürtelrose	antiviral for shingles	Therapy
gürtelrose an der scheide	shingles in vagina	Localization
gürtelrose unterer rücken	shingles lower back	Localization
herpes ohne windpocken	herpes without chickenpox	Causes
herpes zoster herz	herpes zoster heart	Other Diseases
herpes zoster unter der brust	herpes zoster under breast	Localization
schmerzen gürtelrose rücken	pain shingles back	Symptoms, Localization
augen herpes zoster	eyes herpes zoster	Localization
differentialdiagnose herpes zoster	differential diagnosis herpes zoster	Other Diseases
gürtelrose am bein behandlung	shingles on leg therapy	Localization, Therapy
gürtelrose am daumen	shingles on thumb	Localization
gürtelrose in der scheide	shingles in vagina	Localization
gürtelrose mit 35	shingles at 35	Demographics
gürtelrose nur wenn man windpocken hatte	shingles only when one had chickenpox	Causes
gürtelrose offene wunde	shingles open wound	Symptoms
nicht behandelte gürtelrose	untreated shingles	Therapy
paracetamol bei gürtelrose	paracetamol for shingles	Therapy
gürtelrose am auge behandlung	shingles in eye treatment	Localization, Therapy
gürtelrose beckenbereich	shingles pelvic area	Localization
gürtelrose chronische schmerzen	shingles chronic pain	Complications
gürtelrose erwachsene ansteckend	shingles adults contagious	Demographics, Contagiousness
gürtelrose hals nacken	shingles neck nape	Localization
herpes zoster und schwangerschaft	herpes zoster and pregnancy	Demographics
krankmeldung bei gürtelrose	sick leave for shingles	Information
vorboten gürtelrose	prodomal signs shingles	Symptoms
arbeitsunfähig bei gürtelrose	sick leave for shingles	Information
dauer schmerzen gürtelrose	duration pain shingles	Symptoms
folgen gürtelrose kopf	consequences shingles head	Complications, Localization
gürtelrose aber keine schmerzen	shingles but no pain	Symptoms
gürtelrose windpocken herpes	shingles chickenpox herpes	Causes
kommt gürtelrose wieder	does shingles come back again	Information

nebenwirkungen bei gürtelrose	side effects for shingles	Complications
symptome kopfgürtelrose	symptoms shingles head	Symptoms, Localization
varizella zoster ansteckung	varicella zoster contagiousness	Contagiousness
b12 herpes zoster	b12 herpes zoster	Therapy
gibt es gürtelrose ohne bläschen	is there shingles without blisters	Symptoms
gürtelrose am haaransatz	shingles on hairline	Localization
gürtelrose an den fingern	shingles on the fingers	Localization
gürtelrose an der schläfe	shingles on the temples	Localization
gürtelrose im gesicht schmerzen	shingles in face pain	Localization, Symptoms
zoster ophthalmicus augenheilkunde	zoster ophthalmicus ophthalmology	Localization, Therapy
folgeschäden gürtelrose	consequences shingles	Complications
gesichtsrose ohne bläschen	facial shingles no blisters	Localization, Symptoms
gürtelrose ansteckung wenn man schon windpocken hatte	shingles contagious when already had chickenpox	Causes, Contagiousness
gürtelrose lustig	shingles funny	General
gürtelrose unterleib	shingles	Localization
herpes zoster alternativ behandeln	herpes zoster alternative treatment	Therapy
herpes zoster am zeh	herpes zoster on toe	Localization
ist gürtelrose immer schmerzhaft	is shingles always painful	Symptoms
zona zoster kezelese	zona zoster kezelese	General
geht eine gürtelrose von alleine weg	does shingles go away by itself	Therapy
gürtelrose brustbein	shingles sternum	Localization
gürtelrose diagnose und therapie	shingles diagnosis and therapy	General, Therapy
gürtelrose kopfhaut ohne bläschen	shingles scalp without blisters	Localization, Symptoms
gürtelrose kurz hintereinander	recurrence of shingles in quick succession	Information
gürtelrose unterm fuss	shingles under foot	Localization
gürtelrose ursache krebs	shingles cause cancer	Causes
kann man von gürtelrose windpocken bekommen	can one get shingles from chickenpox	Causes
mund gürtelrose	mouth singles	Localization
ansteckung gesichtsrose	contagiousness shingles face	Contagiousness, Localization
gürtelrose chronisch symptome	shingles chronic pain	Complications, Symptoms
gürtelrose milder verlauf	shingles mild disease course	Symptoms
gürtelrose unter dem auge	shingles under eye	Localization

hals gürtelrose	neck shingles	Localization
kann man gürtelrose im gesicht bekommen	can one get shingles in face	Localization
kind hat windpocken kann ich gürtelrose bekommen	child has chickenpox can i get shingles	Contagiousness, Causes
post zoster neuralgie nach jahren	post zoster neuralgia after years	Complications
symptome gürtelrose hüfte	symptoms shingles hips	Symptoms, Localization
bein gürtelrose	leg shingles	Localization
depression nach gürtelrose	depression after shingles	Other Diseases
gürtelrose folgeerkrankungen	shingles complcations	Complications
gürtelrose im kindergarten meldepflichtig	shingles kindergarten reportable	Contagiousness
gürtelrose im rücken	shingles on back	Localization
gürtelrose in der schwangerschaft gefährlich	shingles in pregnancy dangerous	Demographics, Complications
gürtelrose salzwasser	shingles saltwater	Therapy
gürtelrose über dritte ansteckend	shingles infectious through third party	Contagiousness
herpes zoster neurologia	herpes zoster neurology	Symptoms, Complications
muskelschmerzen nach gürtelrose	muscle pain after shingles	Complications
windpocken oder gürtelrose	chickenpox or shingles	Causes
woher gürtelrose	from where shingles	Causes
zoster herpes gesicht	zoster herpes face	Localization
gürtelrose am kopf folgen	shingles on head consequences	Localization, Complications
gürtelrose behandlung und dauer	shingles therapy and duration	Therapy
gürtelrose innenschenkel	shingles inner thigh	Localization
gürtelrose mit 18	shingles with 18	Demographics
herpes zoster s1	herpes zoster s1	Localization
erfahrungsberichte gürtelrose	experiences shingles	Information
gürtelrose nach erkältung	shingles after cold	Causes
herpes zoster vírus	herpes zoster virus	Causes
impfempfehlung gürtelrose	vaccine recommendation shingles	Vaccine
schwache gürtelrose	mild shingles	Symptoms
techniker krankenkasse gürtelrose	techniker krankenkasse shingles	Information
gibt es gürtelrose am fuss	is there shingles on the foot	Localization
gürtelrose ansteckungsgefahr für schwangere	shingles risk of contagion for pregnant women	Contagiousness, Demographics
gürtelrose hochansteckend	shingles highly contagious	Contagiousness

gürtelrose im kopfbereich	shingles on head	Localization
gürtelrose mehrfach bekommen	shingles more than once	Information
post zoster neuralgie schmerztherapie	post zoster neuralgia pain therapy	Complications, Therapy
zoster haut	zoster skin	Symptoms
gürtelrose an armen	shingles on arms	Localization
gürtelrose bei kindern gefährlich	shingles in children dangerous	Demographics, Complications
gürtelrose kinder behandlung	shingles children treatment	Demographics, Therapy
isolation herpes zoster	isolation herpes zoster	Information
ist gürtelrose herpes	is shingles herpes	Other Diseases
nervenschmerzen im kopf nach gürtelrose	nerve pain on head after shingles	Complications, Localization
schmerzen nach gürtelrose hausmittel	pain after shingles home remedy	Complications, Therapy
zinksalbe herpes zoster	zinc ointment herpes zoster	Therapy
fieber gürtelrose	fever shingles	Symptoms
gürtelrose bakterielle infektion	shingles bacterial infection	Complications
gürtelrose beim auge	shingles in eye	Localization
gürtelrose im gesicht anfangsstadium	shingles in face early stage	Localization
herpes zoster mms	herpes zoster mms	Therapy
ist herpes und gürtelrose das gleiche	is herpes and shingles the same thing	Other Diseases
nervenentzündung durch herpes	nerve inflammation because of herpes	Complications
beginnende gürtelrose symptome	early stage shingles symptoms	Symptoms
folgen unbehandelte gürtelrose	complications of untreated shingles	Complications, Therapy
gürtelrose am fuß möglich	shingles on foot possible	Localization
gürtelrose schwangerschaft symptome	shingles pregnancy symptoms	Demographics, Symptoms
gürtelrose ums auge	shingles around the eye	Localization
herpes zoster kinn	herpes zoster chin	Localization
herpes zoster neuralgie symptome	herpes zoster neuralgia symptoms	Symptoms, Complications
ausschlag nach gürtelrose	rash after shinges	Symptoms
gürtelrose bei jungen erwachsenen	shingles in young adults	Demographics
gürtelrose diagnose blut	shingles diagnosis blood	Information
gürtelrose schmerzen behandeln	shingles pain treatment	Symptoms, Therapy
krebs und gürtelrose	cancer and shingles	Other Diseases

nervenentzündung nach gürtelrose	nerve inflammation after shingles	Complications
gürtelrose abklingen	shingles subsiding	General
gürtelrose durch herpes	shingles because of herpes	Causes
gürtelrose für schwangere ansteckend	shingles for pregnant women contagious	Contagiousness, Demographics
gürtelrose leistenschmerz	shingles pain in groin	Localization, Symptoms
gürtelrose nach sonnenbrand	shingles after sunburn	Causes
gürtelrose natürliche heilmittel	shingles natural remedy	Therapy
gürtelrose nervenschmerzen behandeln	shingles nerve pain therapy	Complications, Symptoms, Therapy
gürtelrose nervlich	shingles nerve-related	General
gürtelrose schluckbeschwerden	shingles difficulty swallowing	Symptoms
herpes zoster mit neuralgie	herpes zoster with neuraglai	Symptoms, Complications
kopfrose ansteckungsgefahr	head shingles risk of contagion	Localization, Contagiousness
krankschreiben bei gürtelrose	sick leave for shingles	Information
mevir gürtelrose	mevir shingles	General
ursache gürtelrose stress	causes shingles stress	Causes
b12 bei gürtelrose	b12 for shingles	Therapy
gürtelrose gesicht ursache	shingles face causes	Localization, Causes
gürtelrose infektionskrankheit	shingles infectious disease	General
herpes zoster herpes zoster	herpes zoster herpes zoster	General
nervenschmerzen herpes zoster	nerve pain herpes zoster	Symptoms, Complications
spätfolgen herpes zoster	late complications herpes zoster	Complications
gürtelrose genital	shingles genital	Localization
gürtelrose ohne ausschlag möglich	shingles without rash possible	Symptoms
gürtelrose schmerzfrei	shingles painfree	Symptoms
gürtelrose und rheuma	shingles and rheuma	Other Diseases
herpes durch windpocken	herpes through chickenpox	Causes
herpes zoster symptome ohne ausschlag	herpes zoster symptoms without rash	Symptoms
impfempfehlung herpes zoster	vaccine recommendation herpes zoster	Vaccine
innere gürtelrose ansteckend	internal shingles contagious	Localization, Contagiousness
ist gürtelrose lebensgefährlich	is shingles fatal	Complications
ohr gürtelrose	ear shingles	Localization
vzv v	varicella zoster virus	Causes

akute gürtelrose	akute shingles	General
b12 gürtelrose	b12 shingles	Therapy
chronische nervenschmerzen nach gürtelrose	chronic nerve pain after shingles	Complications
gibt es gürtelrose ohne schmerzen	is there shingles without pain	Symptoms
gürtelrose auf dem kopf symptome	shingles on head symptoms	Localization, Symptoms
gürtelrose therapie naturheilkunde	shingles therapy naturopathy	Therapy
herpes zoster after	herpes zoster anus	Localization
herpes zoster an der hand	herpes zoster on hand	Localization
herpes zoster schmerzen therapie	herpes zoster pain therapy	Therapy, Symptoms
windpocken ansteckung gürtelrose	chickenpox contagious shingles	Causes, Contagiousness
gürtelrose ausschlag ohne schmerzen	shingles rash without pain	Symptoms
gürtelrose nach zeckenbiss	shingles after tick bite	Causes
gürtelrose um die augen	shingles around the eyes	Localization
hautausschlag nach gürtelrose	rash after shingles	Symptoms
herpes zoster alkohol	herpes zoster alcohol	Causes
spätfolgen von gürtelrose	consequences shingles	Complications
